# Predicting outcomes following lower extremity open revascularization using machine learning

**DOI:** 10.1038/s41598-024-52944-1

**Published:** 2024-02-05

**Authors:** Ben Li, Raj Verma, Derek Beaton, Hani Tamim, Mohamad A. Hussain, Jamal J. Hoballah, Douglas S. Lee, Duminda N. Wijeysundera, Charles de Mestral, Muhammad Mamdani, Mohammed Al-Omran

**Affiliations:** 1 Department of Surgery, University of Toronto, Toronto, Canada; 2 Division of Vascular Surgery, St. Michael’s Hospital, Unity Health Toronto, University of Toronto, 30 Bond Street, Toronto, ON M5B 1W8 Canada; 3 Institute of Medical Science, University of Toronto, Toronto, Canada; 4 Temerty Centre for Artificial Intelligence Research and Education in Medicine (T-CAIREM), University of Toronto, Toronto, Canada; 5 School of Medicine, Royal College of Surgeons in Ireland, University of Medicine and Health Sciences, Dublin, Ireland; 6 Data Science & Advanced Analytics, Unity Health Toronto, University of Toronto, Toronto, Canada; 7 Faculty of Medicine, Clinical Research Institute, American University of Beirut Medical Center, Beirut, Lebanon; 8 College of Medicine, Alfaisal University, Riyadh, Kingdom of Saudi Arabia; 9 Division of Vascular and Endovascular Surgery and the Center for Surgery and Public Health, Brigham and Women’s Hospital, Harvard Medical School, Boston, USA; 10 Division of Vascular and Endovascular Surgery, Department of Surgery, American University of Beirut Medical Center, Beirut, Lebanon; 11 Division of Cardiology, Peter Munk Cardiac Centre, University Health Network, Toronto, Canada; 12 Institute of Health Policy, Management and Evaluation, University of Toronto, Toronto, Canada; 13 ICES, University of Toronto, Toronto, Canada; 14 Department of Anesthesia, St. Michael’s Hospital, Unity Health Toronto, Toronto, Canada; 15 Li Ka Shing Knowledge Institute, St. Michael’s Hospital, Unity Health Toronto, Toronto, Canada; 16 Leslie Dan Faculty of Pharmacy, University of Toronto, Toronto, Canada; 17 Department of Surgery, King Faisal Specialist Hospital and Research Center, Riyadh, Kingdom of Saudi Arabia

**Keywords:** Peripheral vascular disease, Prognosis

## Abstract

Lower extremity open revascularization is a treatment option for peripheral artery disease that carries significant peri-operative risks; however, outcome prediction tools remain limited. Using machine learning (ML), we developed automated algorithms that predict 30-day outcomes following lower extremity open revascularization. The National Surgical Quality Improvement Program targeted vascular database was used to identify patients who underwent lower extremity open revascularization for chronic atherosclerotic disease between 2011 and 2021. Input features included 37 pre-operative demographic/clinical variables. The primary outcome was 30-day major adverse limb event (MALE; composite of untreated loss of patency, major reintervention, or major amputation) or death. Our data were split into training (70%) and test (30%) sets. Using tenfold cross-validation, we trained 6 ML models. Overall, 24,309 patients were included. The primary outcome of 30-day MALE or death occurred in 2349 (9.3%) patients. Our best performing prediction model was XGBoost, achieving an area under the receiver operating characteristic curve (95% CI) of 0.93 (0.92–0.94). The calibration plot showed good agreement between predicted and observed event probabilities with a Brier score of 0.08. Our ML algorithm has potential for important utility in guiding risk mitigation strategies for patients being considered for lower extremity open revascularization to improve outcomes.

## Introduction

Peripheral artery disease (PAD) is a chronic atherosclerotic disorder that primarily causes decreased perfusion to the lower extremities, manifesting in claudication, rest pain, and tissue loss^1^. Affecting over 200 million people worldwide, PAD is a major contributor to decreased quality of life, rising health care costs, limb loss, and death^2–5^. Lower extremity open revascularization is a surgical treatment option for PAD that has been recently demonstrated in the BEST-CLI trial to achieve superior outcomes compared to endovascular therapy for chronic limb threatening ischemia (CLTI) in patients with an adequate great saphenous vein conduit^6^. Nevertheless, open revascularization carries a high risk of complications, with major adverse limb event (MALE) or death occurring in over 40% of the surgical group in the BEST-CLI trial after a median follow-up of 2.7 years^6^. Others have shown that over 30% of patients will suffer a major adverse event within 30 days following lower extremity bypass^7^. As a result, the Global Vascular Guidelines recommend careful assessment of surgical risk when considering patients for revascularization^8^.

There are currently no widely used clinical tools to predict adverse events following lower extremity open revascularization. In the research setting, current models are limited to trauma patients^9^, Japanese and Finnish cohorts^10,11^, and prediction of groin wound infections^12^. Furthermore, tools such as the American College of Surgeons (ACS) National Surgical Quality Improvement Program (NSQIP) surgical risk calculator^13^ and Vascular Study Group of New England (VSGNE) Cardiac Risk Index (CRI)^14^ use modelling techniques that require manual input of clinical variables, which deters routine use in busy medical settings^15^. Therefore, there is an important need to develop better and more practical surgical risk prediction tools that overcome existing limitations with automated modelling techniques, inclusion of more geographically diverse cohorts with atherosclerotic disease, and assessment of more clinically relevant outcomes such as MALE or death.

Machine learning (ML) is a rapidly advancing technology that allows computers to learn from data and make predictions^16^. This field has been driven by the explosion of electronic medical record data combined with increasing computational power^17^. Previously, ML has been applied to the ACS NSQIP database to develop algorithms that predict peri-operative complications in a pooled dataset of over 2900 unique procedures, including patients undergoing day surgery to those requiring intensive care unit admission^18^. Given that this cohort represents a heterogeneous surgical population, better predictive performance may be achieved by developing ML algorithms specific to patients undergoing lower extremity open revascularization. In this study, ML was applied to the ACS NSQIP database to predict 30-day MALE or death and other outcomes following lower extremity open revascularization using pre-operative data.

## Methods

### Ethics

All methods were carried out in accordance with the World Medical Association Declaration of Helsinki^19^. Institutional research ethics board review and informed patient consent were not required as the data came from a large, deidentified registry, which is an accepted practice for studies based on ACS NSQIP data^20^.

### Design

We conducted a multicenter retrospective cohort ML-based prognostic study and findings were reported based on the Transparent Reporting of a Multivariable Prediction Model for Individual Prognosis or Diagnosis (TRIPOD) statement^21^.

### Dataset

Created in 2004, the ACS NSQIP database contains demographic, clinical, and 30-day outcomes data on surgical patients across over 700 hospitals in approximately 15 countries worldwide^22^. The information is prospectively collected from electronic health records by trained and certified clinical reviewers and regularly audited by ACS for accuracy^23^. In 2011, targeted NSQIP registries for vascular operations were developed by vascular surgeons, which contain additional procedure-specific variables and outcomes^24^.

### Cohort

All patients who underwent scheduled and unscheduled lower extremity infrainguinal open revascularization for chronic atherosclerotic disease between 2011 and 2021 in the ACS NSQIP targeted vascular database were included. This information was merged with the main ACS NSQIP database for a complete set of generic and procedure-specific variables and outcomes. Patients treated for lower extremity aneurysmal disease, acute limb ischemia, trauma, dissection, or malignancy, as well as those with unreported symptom status (CLTI, claudication, or asymptomatic) or undergoing concurrent major amputation were excluded.

### Features

Thirty-seven pre-operative variables were used as input features for our ML models. Demographic variables included age, sex, body mass index, race, ethnicity, and origin status. Comorbidities included hypertension, diabetes, smoking status, congestive heart failure (CHF), chronic obstructive pulmonary disease (COPD), end stage renal disease (ESRD) requiring dialysis, functional status, and physiologic high-risk factor [defined as at least one of the following: (1) end stage renal disease, (2) age > 80, (3) New York Heart Association CHF class III/IV, (4) left ventricular ejection fraction < 30%, (5) unstable angina within 30 days prior to surgery, or (6) myocardial infarction (MI) within 30 days prior to surgery]. Medications included antiplatelets, statins, and beta blockers. Pre-operative laboratory investigations included serum sodium, blood urea nitrogen (BUN), serum creatinine, albumin, white blood cell count, hematocrit, platelet count, international normalized unit (INR), and partial thromboplastin time (PTT). Limb hemodynamics based on ankle brachial index (ABI), toe pressure, and palpability of pedal pulses, as well as anatomic high-risk factors (defined as a prior bypass or endovascular intervention involving the currently treated segment) were recorded. Concurrent procedures recorded included minor amputation (below the ankle) and endovascular iliac or infrainguinal revascularization. Other pre-procedural characteristics recorded were symptom status [asymptomatic, claudication, or CLTI (defined as rest pain or tissue loss)], primary procedure including inflow, outflow, and conduit, urgency of surgery (elective, urgent, or emergent), and American Society of Anesthesiologists (ASA) class. A complete list of features and definitions can be found in Supplementary Table 1.

### Outcomes

The primary outcome was 30-day MALE (composite of untreated loss of patency, major reintervention, or major amputation) or death. Untreated loss of patency was defined as a loss of graft patency on imaging or physical exam with no subsequent open or endovascular revascularization procedure. Major reintervention was defined as a new or revision lower extremity bypass, interposition graft revision, or bypass graft thrombectomy/thrombolysis. Major amputation was defined as a transtibial or more proximal amputation on the ipsilateral leg. Death was defined as all-cause mortality. This composite outcome was chosen because it is frequently reported as a primary outcome in landmark studies, including the BEST-CLI trial^6^.

Thirty-day secondary outcomes included individual components of the primary outcome, major adverse cardiovascular event (MACE), individual components of MACE, wound complication, bleeding requiring transfusion or secondary procedure, other morbidity, non-home discharge, and unplanned readmission. MACE was defined as a composite of MI (ischemic electrocardiogram changes, troponin elevation, or physician/advanced provider diagnosis), stroke (motor, sensory, or cognitive dysfunction persisting for > 24 h in the setting of suspected stroke), or death. Wound complication was defined as a non-healing or open wound at the surgical incision, dehiscence, or cellulitis. Other morbidity was defined as a composite of pneumonia, unplanned reintubation, pulmonary embolism (PE), failure to wean from ventilator (cumulative time of ventilator-assisted respirations > 48 h), acute kidney injury (AKI; rise in creatinine of > 2 mg/dL from pre-operative value or requirement of dialysis in a patient who did not require dialysis pre-operatively), urinary tract infection (UTI), cardiac arrest, deep vein thrombosis (DVT) requiring therapy, *Clostridium difficile* infection, sepsis, or septic shock. Non-home discharge was defined as discharge to rehabilitation, skilled care, or other facility. These outcomes are defined by the ACS NSQIP data dictionary^25^.

### Model development

Six ML models were trained to predict 30-day primary and secondary outcomes: Extreme Gradient Boosting (XGBoost), random forest, Naïve Bayes classifier, radial basis function (RBF) support vector machine (SVM), multilayer perceptron (MLP) artificial neural network (ANN) with a single hidden layer, sigmoid activation function, and cross-entropy loss function, and logistic regression. These were chosen because they demonstrate the best performance for predicting surgical outcomes in the literature^26–28^. XGBoost is a gradient-boosted decision-tree-based ensemble model that is highly effective at regression and classification predictive modelling^29^. Random forest is an ensemble learning method that operates through multiple decision trees^30^. Naïve Bayes classifiers apply Bayes’ theorem to generate highly accurate predictions in high-dimensional datasets^31^. SVM’s can find hyperplanes in dimensional space to distinctly separate data points and achieve binary classification^32^. Neural networks resemble biological neurons and consist of an input, hidden, and output layer, capable of making meaningful predictions from complex information^33^. Logistic regression is a traditional statistical method used to model the relationship between independent and dependent variables, assuming a linear correlation between the predictors and logit of the outcome, as well as a lack of multicollinearity between explanatory variables^34^. The advantage of newer ML techniques over logistic regression is that they apply more advanced analytics to better model complex, multicollinear relationships between predictors and outcomes^35^. Nonlinear associations are common in health care data as patient trajectories are often influenced by many clinical, demographic, and systems-level factors^36^. Logistic regression was therefore used as the baseline comparator to assess relative model performance because it is the most common modelling technique used in traditional risk prediction tools^37^.

Our data were split into training (70%) and test (30%) sets^38^. Ten-fold cross-validation and grid search were performed on the training set to find optimal hyperparameters for each ML model^39,40^. Preliminary analysis of our data demonstrated that the primary outcome was uncommon, occurring in 2349/24,309 (9.7%) of patients in our cohort. To improve class balance, Random Over-Sample Examples (ROSE) was applied^41^. ROSE uses smoothed bootstrapping to draw new samples from the feature space neighbourhood around the minority class and is a commonly used method to support predictive modelling of rare events^41^. The models were then evaluated on test set data and ranked based on the primary discriminatory metric of AUROC. Our best performing model was XGBoost, which had the following optimized hyperparameters for our dataset: number of rounds = 100, maximum tree depth = 6, learning rate = 0.3, gamma = 0, column sample by tree = 1, minimum child weight = 1, subsample = 1. The process for selecting these hyperparameters through grid search and cross validation is detailed in Supplementary Table 2. Once we identified XGBoost as the best performing ML model for the primary outcome, we trained the algorithm to predict secondary outcomes.

### Statistical analysis

Baseline demographic and clinical characteristics for patients with vs. without 30-day MALE or death were summarized as means (standard deviation), medians (interquartile range), or number (proportion). Differences in characteristics between outcome groups were assessed using independent t-test for continuous variables or chi-square test for categorical variables. Statistical significance was set at two-tailed* p *< 0.05.

The primary metric for assessing model performance was AUROC (95% CI), a validated method to assess discriminatory ability that considers both sensitivity and specificity^42^. Secondary performance metrics were accuracy, sensitivity, specificity, positive predictive value (PPV), and negative predictive value (NPV). To further assess model performance, we plotted a calibration curve and calculated the Brier score, a measurement of the agreement between predicted and observed event probabilities^43^. In the final model, feature importance was determined by ranking the top 10 predictors based on the variable importance score (gain), a measure of the relative impact of individual covariates in contributing to an overall prediction^44^. Feature importance was determined for the overall cohort, CLTI patients, and asymptomatic/claudication groups. To assess model robustness on various populations, we performed subgroup analysis of predictive performance based on age (under vs. over 70 years), sex (male vs. female), race (White vs. non-White), ethnicity (Hispanic vs. Non-Hispanic), symptom status (CLTI vs. asymptomatic/claudication), procedure type (femoropopliteal bypass vs. femoral to tibial/pedal bypass vs. popliteal to tibial/pedal bypass vs. femoral endarterectomy/profundoplasty), and urgency (urgent/emergent vs. elective).

Based on a validated sample size calculator for clinical prediction models, to achieve a minimum AUROC of 0.7 with an outcome rate of ~ 10% and 37 input features, the minimum sample size required is 6960 patients with 696 events^45,46^. Our cohort of 24,309 patients with 2349 primary events meets this sample size requirement. There was less than 5% missing data for variables of interest; therefore, complete-case analysis was applied whereby only non-missing covariates for each patient were considered^47^. This has been demonstrated to be a valid analytical method for datasets with small amounts of missing data (< 5%) and reflects predictive modelling of real-world data, which inherently includes missing information^48,49^. All analyses were performed in R version 4.2.1^50^ with the following packages: caret^51^, xgboost^52^, ranger^53^, naivebayes^54^, e1071^55^, nnet^56^, and pROC^57^.

## Results

### Patients and events

From an initial cohort of 25,318 patients who underwent lower extremity open revascularization in the NSQIP targeted database between 2011 and 2021, we excluded 1,009 patients for the following reasons: treatment for lower extremity aneurysmal disease (n = 669), acute limb ischemia (n = 24), trauma (n = 4), or dissection (n = 2), unreported symptom status (n = 306), and concurrent major amputation (n = 4). Overall, we included 24,309 patients. The primary outcome of 30-day MALE or death occurred in 2349 (9.3%) patients. The 30-day secondary outcomes occurred in the following distribution: untreated loss of patency (n = 457 [1.9%]), major reintervention (n = 1,11 [4.9%]), major amputation (n = 689 [2.8%]), death (n = 547 [2.3%]), MACE (n = 1,346 [5.5%]), MI (n = 771 [3.2%]), stroke (n = 225 [0.9%]), wound complication (n = 3241 [13.3%]), bleeding requiring transfusion or secondary procedure (n = 4041 [16.6%]), other morbidity (n = 1799 [7.4%]; composite of pneumonia (n = 343), unplanned reintubation (n = 380), PE (n = 62), failure to wean from ventilator (n = 223), AKI (n = 123), UTI (n = 328), cardiac arrest (n = 230), DVT (n = 185), sepsis (n = 475), septic shock (n = 190), *Clostridium difficile* infection (n = 85)), non-home discharge (n = 6954 [28.6%]), and unplanned readmission (n = 3621 [14.9%]).

### Pre-operative demographic and clinical characteristics

Compared to patients without a primary outcome, those who developed 30-day MALE or death were older and more likely to be female, Black, Hispanic, and transferred from another hospital, with a greater proportion residing in nursing homes. They were also more likely to have insulin dependent diabetes, CHF, ESRD requiring dialysis, and at least 1 physiologic high-risk factor. Functionally, patients with 30-day MALE or death were more likely to be partially or totally dependent. Despite being at higher cardiovascular risk, patients with 30-day MALE or death were less likely to receive antiplatelets. Notable differences in laboratory investigations included patients with 30-day MALE or death having higher levels of creatinine and BUN. Patients with a primary outcome were more likely to have an ABI ≤ 0.39 and a previous bypass or endovascular intervention involving the currently treated segment, with a greater proportion undergoing a concurrent minor amputation or endovascular infrainguinal revascularization. Patients with 30-day MALE or death were more likely to have CLTI, undergo a bypass to a tibial/pedal target, receive urgent/emergent surgery, and be ASA class 4 or higher (Table 1).

**Table 1:** Pre-operative demographic and clinical characteristics of patients undergoing lower extremity open revascularization with and without major adverse limb event or death at 30 days.

	Absence of MALE or death at 30 days (n = 21,960)	Presence of MALE or death at 30 days (n = 2349)	*P*
Demographics			
Age, years, mean (SD)	67.7 (9.9)	68.4 (10.7)	< 0.001
Female	7240 (33.0)	871 (37.1)	< 0.001
BMI, kg/m^2^, mean (SD)	27.8 (5.8)	27.7 (6.3)	0.29
Race			
White	14,165 (64.5)	1,405 (59.8)	< 0.001
Black or African American	3595 (16.4)	454 (19.3)	
American Indian or Alaskan Native	72 (0.3)	6 (0.3)	
Native Hawaiian or Other Pacific Islander	18 (0.08)	0	
Asian	178 (0.8)	19 (0.8)	
Other	15 (0.07)	3 (0.1)	
Unknown or not reported	3917 (17.8)	462 (19.7)	
Hispanic ethnicity	1072 (4.9)	130 (5.5)	0.004
Origin status			
Transferred from another hospital	1534 (7.0)	277 (11.8)	< 0.001
Home	19,768 (90.0)	1,956 (83.3)	
Nursing home	523 (2.4)	90 (3.8)	
Other facility	128 (0.6)	25 (1.1)	
Unknown	7 (0.03)	1 (0.04)	
Comorbidities			
Hypertension	17,924 (81.6)	1908 (81.2)	0.66
Diabetes			
Non-insulin dependent	4228 (19.3)	417 (17.8)	0.15
Insulin dependent	5993 (27.3)	690 (29.4)	0.03
Current smoker	9404 (42.8)	990 (42.1)	0.69
Congestive heart failure	762 (3.5)	138 (5.9)	< 0.001
Chronic obstructive pulmonary disease	2754 (12.5)	315 (13.4)	0.24
Dialysis	1205 (5.5)	252 (10.7)	< 0.001
Functional status			
Independent	20,463 (93.2)	2057 (87.6)	< 0.001
Partially dependent	1310 (6.0)	247 (10.5)	
Totally dependent	106 (0.5)	27 (1.2)	
Unknown	81 (0.4)	18 (0.8)	
Physiologic high-risk factor^a^	4578 (20.8)	735 (31.3)	< 0.001
Medications			
Antiplatelet	17,947 (81.7)	1814 (77.2)	< 0.001
Statin	15,900 (72.4)	1666 (70.9)	0.08
Beta blocker	12,394 (56.4)	1368 (58.2)	0.11
Laboratory investigations			
Sodium, mmol/L, mean (SD)	138.0 (3.4)	137.0 (3.6)	< 0.001
BUN, mmol/L, mean (SD)	56.2 (31.6)	60.1 (39.1)	< 0.001
Creatinine, umol/L, median (IQR)	88.4 (70.7–115.0)	91.1 (70.7–124.0)	< 0.001
Albumin, g/L, mean (SD)	26.3 (10.2)	26.3 (9.6)	0.92
White blood cell count, cells/mm^3^, mean (SD)	8.4 (4.4)	9.3 (4.6)	< 0.001
Hematocrit, L/L (%), mean (SD)	37.4 (6.2)	35.9 (6.5)	< 0.001
Platelet count, 10^9^/L, mean (SD)	251.0 (92.3)	265.0 (111.0)	< 0.001
INR, mean (SD)	1.1 (0.3)	1.2 (0.4)	< 0.001
PTT, sec, mean (SD)	34.0 (13.9)	37.0 (17.2)	< 0.001
Anatomy/hemodynamics			
Limb hemodynamics			
ABI ≥ 1.3 and toe pressure ≥ 30 mmHg	164 (0.7)	20 (0.9)	< 0.001
ABI ≥ 1.3 and toe pressure < 30 mmHg	158 (0.7)	24 (1.0)	
ABI ≥ 1.3 and no toe pressure recorded	341 (1.6)	48 (2.0)	
ABI 0.90 – 1.29	599 (2.7)	60 (2.6)	
ABI 0.40 – 0.89	6861 (31.2)	502 (21.4)	
ABI ≤ 0.39	4060 (18.5)	502 (21.4)	
ABI not recorded and palpable pedal pulse	941 (4.3)	78 (3.3)	
ABI not recorded and pedal pulse not palpable	3947 (18.0)	567 (24.1)	
Not documented	4889 (22.3)	548 (23.3)	
Anatomic high-risk factor			
Prior bypass involving currently treated segment	4655 (21.2)	670 (28.5)	< 0.001
Prior endovascular intervention involving currently treated segment	4013 (18.3)	419 (17.8)	
None/not documented	13,292 (60.5)	1,260 (53.6)	
Concurrent procedures			
Minor amputation	870 (4.0)	119 (5.1)	0.01
Endovascular iliac revascularization	789 (3.6)	92 (3.9)	0.46
Endovascular infrainguinal revascularization	499 (2.3)	78 (3.3)	0.002
Other pre-procedural characteristics			
Symptom status			
Asymptomatic	431 (2.0)	21 (0.9)	< 0.001
Claudication	5808 (26.4)	296 (12.6)	
Chronic limb threatening ischemia: rest pain	6408 (29.2)	808 (34.4)	
Chronic limb threatening ischemia: tissue loss	9313 (42.4)	1,224 (52.1)	
Primary procedure			
Femoropopliteal bypass with single segment saphenous vein	6928 (31.5)	550 (23.4)	< 0.001
Femoropopliteal bypass with prosthetic, spliced vein, or composite	5649 (25.7)	450 (19.2)	
Femoral to tibial/pedal bypass with single segment saphenous vein	4337 (19.7)	575 (24.5)	
Femoral to tibial/pedal bypass with prosthetic, spliced vein, or composite	2348 (10.7)	433 (18.4)	
Popliteal to tibial/pedal bypass with single segment saphenous vein	1549 (7.1)	195 (8.3)	
Popliteal to tibial/pedal bypass with prosthetic, spliced vein, composite, or non-saphenous conduit	460 (2.1)	67 (2.9)	
Femoral endarterectomy	257 (1.2)	35 (1.5)	
Profundoplasty	69 (0.3)	5 (0.2)	
Not documented/other	363 (1.7)	39 (1.7)	
Urgency			
Elective	14,632 (66.6)	1,164 (49.6)	< 0.001
Urgent	6050 (27.6)	887 (37.8)	
Emergent	1267 (5.8)	295 (12.6)	
ASA class			
1	37 (0.2)	5 (0.2)	< 0.001
2	813 (3.7)	70 (3.0)	
3	15,594 (71.0)	1449 (61.7)	
4	5476 (24.9)	815 (34.7)	
5	15 (0.07)	8 (0.3)	
Not reported	25 (0.1)	2 (0.09)	

Values are reported as No. (%) unless otherwise indicated.

MALE (major adverse limb event), BMI (body mass index), BUN (blood urea nitrogen), INR (international normalized ratio), PTT (partial thromboplastin time), ABI (ankle brachial index), ASA (American Society of Anesthesiologists), SD (standard deviation).

^a^At least 1 of the following: (1) end stage renal disease, (2) age > 80, (3) New York Heart Association congestive heart failure class III/IV, (4) left ventricular ejection fraction < 30%, (5) unstable angina within 30 days prior to surgery, or (6) myocardial infarction within 30 days prior to surgery.

### Model performance

Of the 6 ML models evaluated on test set data for predicting 30-day MALE or death following lower extremity open revascularization, XGBoost had the best performance with an AUROC (95% CI) of 0.93 (0.92–0.94) compared to random forest [0.92 (0.91–0.93)], Naïve Bayes [0.87 (0.86–0.88)], RBF SVM [0.85 (0.84–0.86)], MLP ANN [0.80 (0.78–0.82)], and logistic regression [0.63 (0.61–0.65)]. The other performance metrics of XGBoost were the following: accuracy 0.86 (95% CI 0.85–0.87), sensitivity 0.84, specificity 0.89, PPV 0.90, and NPV 0.83 (Table 2).

**Table 2:** Model performance on test set data for predicting 30-day major adverse limb event or death following lower extremity open revascularization using pre-operative features.

	AUROC (95% CI)	Accuracy (95% CI)	Sensitivity	Specificity	PPV	NPV
XGBoost	0.93 (0.92–0.94)	0.86 (0.85–0.87)	0.84	0.89	0.90	0.83
Random forest	0.92 (0.91–0.93)	0.85 (0.84–0.86)	0.84	0.86	0.86	0.83
Naïve bayes	0.87 (0.86–0.88)	0.85 (0.84–0.86)	0.83	0.85	0.86	0.82
RBF SVM	0.85 (0.84–0.86)	0.77 (0.75–0.79)	0.75	0.80	0.83	0.71
MLP ANN	0.80 (0.78–0.82)	0.73 (0.70–0.75)	0.71	0.75	0.79	0.69
Logistic regression	0.63 (0.61–0.65)	0.58 (0.56–0.60)	0.55	0.71	0.60	0.56

Abbreviations: XGBoost (Extreme Gradient Boosting), AUROC (area under the receiver operating characteristic curve), CI (confidence interval), PPV (positive predictive value), NPV (negative predictive value), RBF SVM (radial basis function support vector machine), MLP ANN (multilayer perceptron artificial neural network).

For 30-day secondary outcomes, XGBoost achieved the following AUROC’s (95% CI): untreated loss of patency [0.90 (0.89–0.91)], major reintervention [0.91 (0.89–0.93)], major amputation [0.95 (0.94–0.96)], death [0.96 (0.95–0.96)], MACE [0.93 (0.92–0.94)], MI [0.88 (0.87–0.89)], stroke [0.91 (0.90–0.92)], wound complication [0.90 (0.88–0.92)], bleeding requiring transfusion or secondary procedure [0.92 (0.91–0.93)], other morbidity [0.91 (0.89–0.92)], non-home discharge [0.95 (0.95–0.96)], and unplanned readmission [0.87 (0.86–0.89)] (Table 3).

**Table 3:** XGBoost performance on test set data for predicting 30-day primary and secondary outcomes following lower extremity open revascularization using pre-operative features.

	AUROC (95% CI)	Accuracy (95% CI)	Sensitivity	Specificity	PPV	NPV
MALE or death	0.93 (0.92–0.94)	0.86 (0.85–0.87)	0.84	0.89	0.90	0.83
Untreated loss of patency	0.90 (0.89–0.91)	0.83 (0.81–0.84)	0.81	0.85	0.86	0.79
Major reintervention	0.91 (0.89–0.93)	0.84 (0.82–0.86)	0.83	0.85	0.87	0.80
Major amputation	0.95 (0.94–0.96)	0.88 (0.87–0.89)	0.86	0.90	0.91	0.85
Death	0.96 (0.95–0.96)	0.89 (0.87–0.90)	0.87	0.90	0.90	0.87
MACE	0.93 (0.92–0.94)	0.85 (0.84–0.86)	0.84	0.86	0.88	0.85
Myocardial infarction	0.88 (0.87–0.89)	0.80 (0.79–0.82)	0.79	0.81	0.82	0.78
Stroke	0.91 (0.90–0.92)	0.83 (0.81–0.84)	0.82	0.84	0.85	0.80
Wound complication	0.90 (0.88–0.92)	0.82 (0.83–0.87)	0.82	0.83	0.86	0.79
Bleeding requiring transfusion or secondary procedure	0.92 (0.91–0.93)	0.84 (0.82–0.85)	0.82	0.86	0.87	0.81
Other morbidity	0.91 (0.89–0.92)	0.82 (0.81–0.84)	0.81	0.84	0.85	0.79
Non-home discharge	0.95 (0.95–0.96)	0.89 (0.88–0.90)	0.86	0.92	0.92	0.85
Unplanned readmission	0.87 (0.86–0.89)	0.80 (0.78–0.82)	0.80	0.77	0.77	0.81

XGBoost (Extreme Gradient Boosting), AUROC (area under the receiver operating characteristic curve), CI (confidence interval), PPV (positive predictive value), NPV (negative predictive value), MALE (major adverse limb event; composite of untreated loss of patency, major reintervention, or major amputation), MACE (major adverse cardiovascular event; composite of myocardial infarction, stroke, or death).

**Figure 1:**
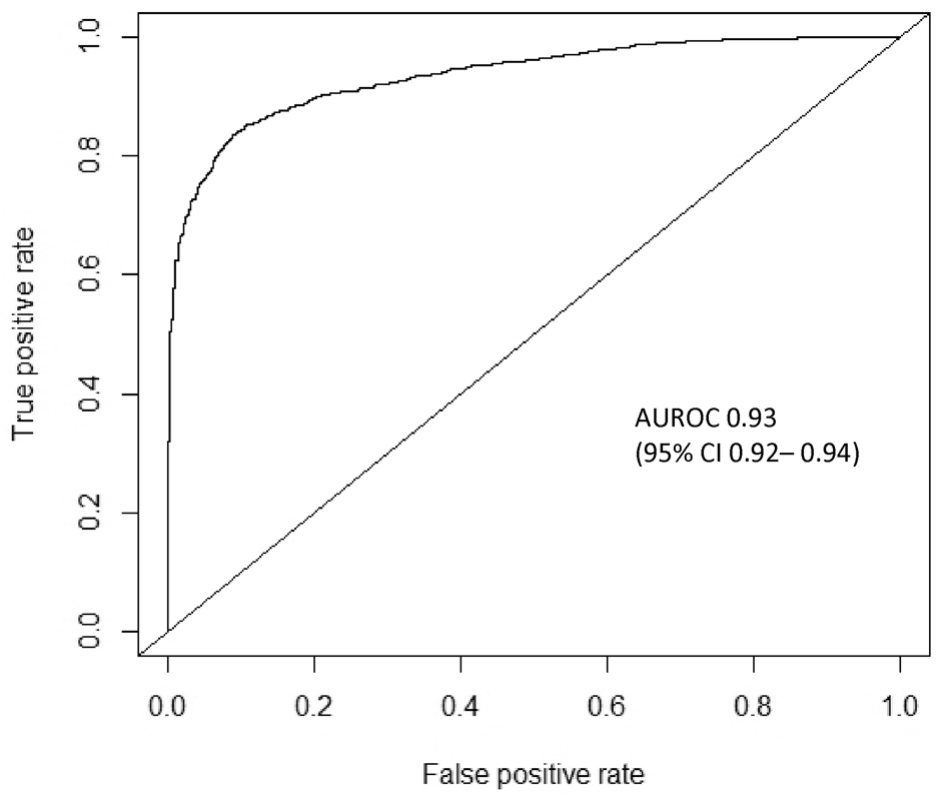
Receiver operating characteristic curve for predicting 30-day major adverse limb event or death following lower extremity open revascularization using Extreme Gradient Boosting (XGBoost) model. AUROC (area under the receiver operating characteristic curve), CI (confidence interval).

**Figure 2:**
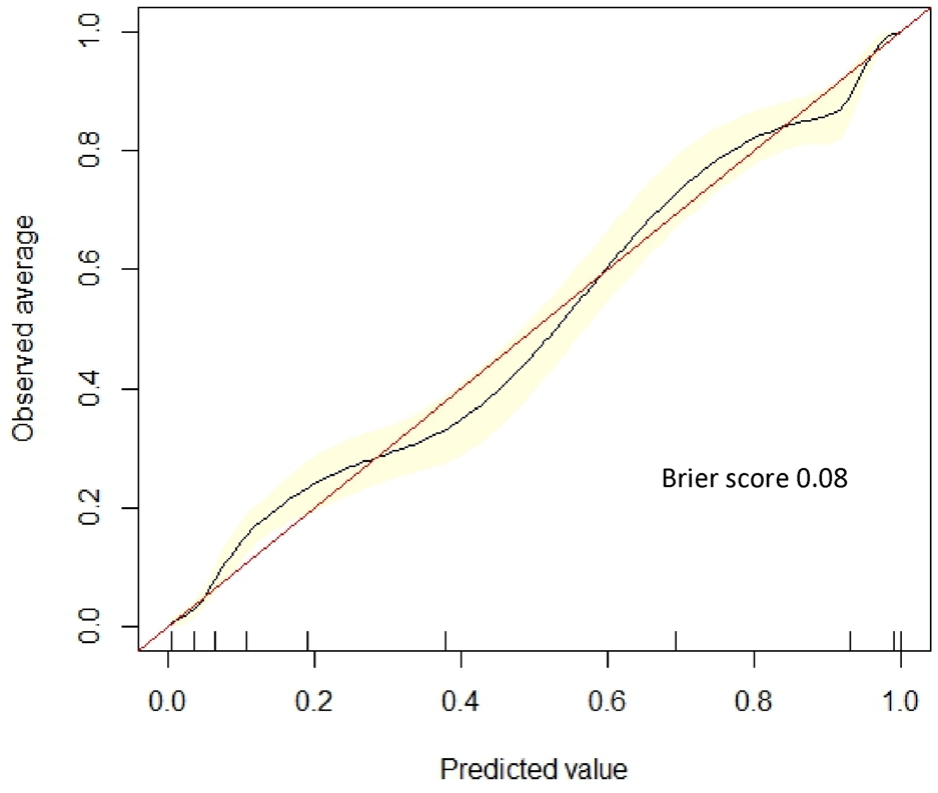
Calibration plot with Brier score for predicting 30-day major adverse limb event or death following lower extremity open revascularization using Extreme Gradient Boosting (XGBoost) model.

**Figure 3:**
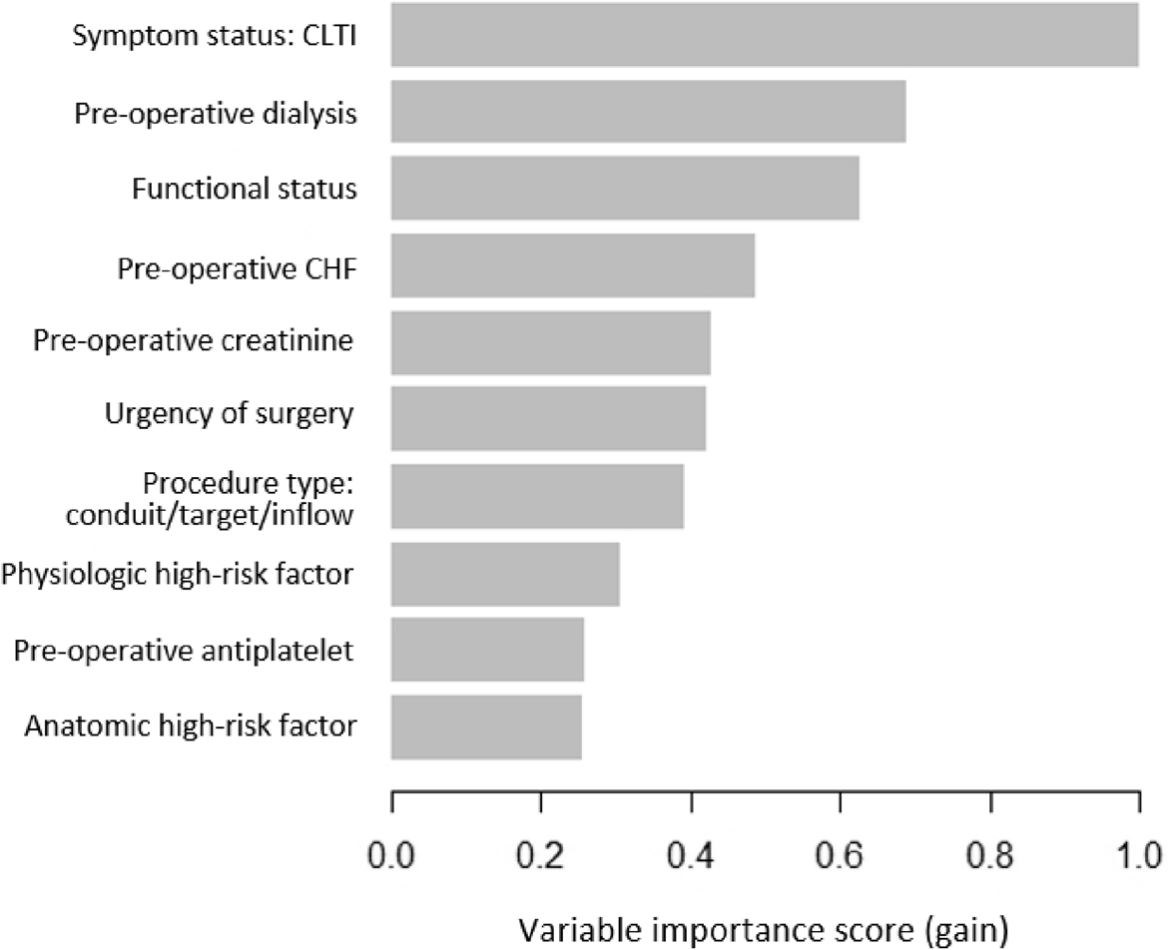
Variable importance scores (gain) for the top 10 predictors of 30-day major adverse limb event or death following lower extremity open revascularization in the Extreme Gradient Boosting (XGBoost) model. Abbreviations: CLTI (chronic limb threatening ischemia), CHF (congestive heart failure).

The ROC curve for prediction of 30-day MALE or death using XGBoost is demonstrated in Fig. 1. Our model achieved good calibration with a Brier score of 0.08, indicating excellent agreement between predicted and observed evented probabilities (Fig. 2). The top 10 predictors of 30-day MALE or death in our XGBoost model were the following: (1) symptom status: CLTI, (2) pre-operative dialysis, (3) functional status, (4) pre-operative CHF, (5) pre-operative creatinine, (6) urgency of surgery, (7) procedure type: conduit/target/inflow, (8) physiologic high-risk factor, (9) pre-operative antiplatelet, and (10) anatomic high-risk factor (Fig. 3). On subgroup analysis based on symptom status, 9/10 of the most important predictive features were the same for patients with CLTI and those who were asymptomatic or had claudication, with the two most important predictors being functional status and pre-operative dialysis for both groups (Supplementary Fig. 1).

### Subgroup analysis

Our XGBoost model performance for predicting 30-day MALE or death remained excellent on subgroup analysis of specific demographic and clinical populations with the following AUROC’s (95% CI): age < 70 [0.93 (0.92–0.94)] and age > 70 [0.94 (0.93–0.95)] (Supplementary Fig. 2), males [0.94 (0.93–0.95)] and females [0.93 (0.91–0.94)] (Supplementary Fig. 3), White patients [0.93 (0.92–0.94)] and non-White patients [0.93 (0.92–0.94)] (Supplementary Fig. 4), Hispanic patients [0.93 (0.92–0.94)] and non-Hispanic patients [0.93 (0.92–0.94)] (Supplementary Fig. 5), CLTI patients [0.93 (0.92–0.94)] and asymptomatic/claudication groups [0.94 (0.93–0.95)] (Supplementary Fig. 6), femoropopliteal bypass [0.94 (0.93–0.95)], femoral to tibial/pedal bypass [0.93 (0.92–0.94)], popliteal to tibial/pedal bypass [0.93 (0.91–0.95)], and femoral endarterectomy/profundoplasty [0.93 (0.89–0.96)] (Supplementary Fig. 7), and urgent/emergent surgery [0.94 (0.93–0.95)] and elective surgery [0.93 (0.92–0.94)] (Supplementary Fig. 8).

## Discussion

### Summary of findings

Using data from the ACS NSQIP targeted vascular files between 2011 and 2021 consisting of 24,309 patients who underwent lower extremity open revascularization for atherosclerotic disease, we developed ML models that accurately predict 30-day MALE or death with an AUROC of 0.93 using pre-operative variables. Furthermore, our algorithms predicted 30-day untreated loss of patency, major reintervention, major amputation, death, MACE, MI, stroke, wound complication, bleeding, other morbidity, non-home discharge, and readmission with AUROC’s between 0.87 and 0.96. There were several other key findings. First, patients who develop 30-day MALE or death represent a high-risk population with several predictive features at the pre-operative stage. Specifically, they are older with more comorbidities, have poorer functional status, and are more likely to have high-risk physiologic and anatomic factors. In addition, a greater proportion of patients with 30-day MALE or death had CLTI, underwent tibial/pedal bypasses, and required concurrent minor amputation or endovascular revascularization. Despite these differences, they were less likely to receive optimal medical therapy including antiplatelets. This represents an important opportunity to improve medical management of PAD patients. Second, we trained 6 ML models to predict 30-day MALE or death using pre-operative features and showed that XGBoost achieved the best performance. Our model was well-calibrated, achieving a Brier score of 0.08, and remained robust on subgroup analysis based on age, sex, race, ethnicity, symptom status, procedure type, and urgency of surgery. Finally, we identified the top 10 predictors of 30-day MALE or death in our ML models. These features can be used by clinicians to identify factors that contribute to risk predictions, thereby guiding patient selection and pre-operative optimization. For example, patients with modifiable high-risk factors could be further evaluated and optimized through pre-operative consultations with anesthesiologists or cardiologists to mitigate adverse events^58,59^. Overall, we have developed a robust ML-based surgical risk prediction tool that can help guide clinical decision-making to improve outcomes and reduce costs from complications, reinterventions, and readmissions associated with lower extremity open revascularization.

### Comparison to existing literature

Bertges et al. developed the VSGNE CRI to predict in-hospital major adverse cardiac events in patients undergoing major vascular procedures including lower extremity bypass, carotid endarterectomy, and aortic aneurysm repair^14^. Using logistic regression, their model achieved an AUROC of 0.71^14^. Applying ML techniques to a more up-to-date cohort specifically consisting of patients undergoing lower extremity open revascularization, we achieved better performance with an AUROC of 0.93.

Bonde et al. trained ML algorithms on a cohort of NSQIP patients undergoing > 2900 unique procedures to predict peri-operative complications, achieving AUROC’s of 0.85–0.88^18^. Given that patients undergoing lower extremity open revascularization for atherosclerotic disease represent a unique population with a high number of vascular comorbidities, the applicability of general surgical risk prediction tools may be limited. By developing ML algorithms specific to patients undergoing lower extremity open revascularization, we achieved AUROC’s > 0.90. Additionally, we included limb- and graft-related outcomes such as major amputation, major reintervention, and untreated loss of patency, which are of clinical importance to vascular surgeons. Therefore, there is value in building procedure-specific ML models, which can increase accuracy and clinical applicability.

Prediction models specific to patients undergoing lower extremity revascularization remain limited. Miyata et al. (2021) applied logistic regression to predict 30-day major amputation or death in a cohort of 2906 patients identified through the Japan Critical Limb Ischemia Database, achieving an AUROC of 0.82^10^. Using a cohort of 24,309 patients in the multi-national NSQIP database, we achieved an AUROC > 0.90 for predicting MALE or death with ML techniques. This demonstrates the benefits of applying advanced analytical techniques to larger and more diverse datasets.

### Explanation of findings

There are several explanations for our findings. First, patients who develop MALE or death following lower extremity revascularization represent a high-risk group with multiple vascular risk factors, as corroborated by previous literature^60^. The use of antiplatelet therapy is a Grade 1A recommendation by multiple societal guidelines for all patients regardless of symptom status (asymptomatic, claudication, or CLTI)^8,61–63^, yet patients who developed MALE or death in our cohort were less likely to receive antiplatelets. The suboptimal rates of best medical therapy for PAD patients are further demonstrated in the recently published BEST-CLI trial^6^. Therefore, there are important opportunities to improve care for patients by understanding their perioperative risk and medically optimizing them prior to revascularization. Second, our ML models performed better than existing tools for several reasons. Compared to traditional logistic regression, advanced ML techniques can better model non-linear, complex relationships between inputs and outputs^64,65^. This is especially important in health care data, as patient outcomes can be influenced by many demographic and clinical factors^66^. Our best performing algorithm was XGBoost, which has unique advantages including avoiding overfitting and faster computing while maintaining precision^67–69^. Furthermore, XGBoost works well with structured data, which may explain why it performed better than more complex algorithms such as neural networks on our dataset^70^. Third, our model performance remained robust on subgroup analysis of specific demographic and clinical populations. This is an important finding given that algorithm bias against underrepresented populations is a significant issue in ML studies^71^. We were likely able to avoid these biases due to the excellent capture of sociodemographic data by ACS NSQIP, a multi-national database that includes diverse patient populations^72,73^. Fourth, a small proportion of patients in our cohort underwent lower extremity open revascularization for asymptomatic disease (< 2%). The reasons for these interventions are unclear from our dataset but may be related to revisions for hemodynamically significant stenoses of previous revascularization procedures, patient preference, poor adherence to guideline-directed revascularization, or coding errors^74^.

### Implications

Our ML models can be used to guide clinical decision-making in several ways. Pre-operatively, a patient predicted to be at high risk of an adverse outcome should be further assessed in terms of modifiable and non-modifiable factors^75^. Patients with significant non-modifiable risk of adverse outcomes following open surgical revascularization may benefit from careful considerations of alternative options including medical management alone or less invasive endovascular therapy^76,77^. Those with modifiable risks should be referred to anesthesiologists, cardiologists, and/or internal medicine specialists for further evaluation^58,59^. Intra-operatively, risk predictions may inform decisions regarding anesthetic techniques such as neuraxial vs. general anesthesia^78^. At the post-operative stage, patients at high risk of adverse events may benefit from close monitoring in the intensive care unit^79^. Additionally, patients at high risk of non-home discharge or readmission should receive early support from allied health professionals to optimize safe discharge planning^80^. These peri-operative decisions guided by our ML models have the potential to improve outcomes and reduce costs by mitigating adverse events.

The programming code used to develop our ML models is publicly available through GitHub, a web-based platform that offers a free and integrated environment for hosting source code, documentation, and project-related web content for open-source projects^81^. These tools can be used by any clinician involved in the peri-operative management of patients being considered for lower extremity open revascularization. On a systems-level, our models can be readily implemented by the > 700 centres that currently participate in ACS NSQIP worldwide. They also have potential for use at non-NSQIP sites, as the input features are commonly captured variables for the routine care of vascular surgery patients^82^. Given the challenges of deploying prediction models into clinical practice, consideration of principles of implementation science is critical^83^. Our ML models have the advantage of providing automated risk predictions using many input variables, thereby improving practicality in busy clinical settings compared to traditional risk predictors that generally require manual input of variables^13^. Specifically, our algorithms were built to autonomously extract a patient’s prospectively collected NSQIP data to make risk predictions. Ongoing efforts to link NSQIP data to electronic health records has the potential to increase the clinical utility of our model and further support fully automated risk predictions^84,85^. We advocate for dedicated health care data analytics teams at the institution level, as their significant benefits have been previously demonstrated and model implementation can be facilitated by these experts^86,87^. Through this study, we have also provided a framework for the development of robust ML models that predict lower extremity open revascularization outcomes, which can be applied by individual centers for their specific patient populations.

### Limitations

Our study has several limitations. First, our models were developed using ACS NSQIP data. Future studies should assess whether performance can be generalized to institutions that do not participate in ACS NSQIP. Second, the ACS NSQIP database captures 30-day outcomes. Evaluation of ML models on data sources with longer follow-up would augment our understanding of long-term surgical risk. Third, our dataset did not capture low-dose rivaroxaban use. Given that the VOYAGER^60^ and COMPASS^88^ trials demonstrated the cardiovascular and limb benefits of low-dose rivaroxaban, future prediction models on datasets that capture this variable may improve performance. Fourth, our models are limited to patients undergoing open revascularization. Future prediction tools for outcomes following endovascular therapy would be helpful to further guide clinical decision-making.

## Conclusions

In this study, we used the ACS NSQIP database to develop robust ML models that pre-operatively predict 30-day MALE or death following lower extremity open revascularization for atherosclerotic disease with excellent performance (AUROC 0.93). Our models also predicted untreated loss of patency, major reintervention, major amputation, death, MACE, MI, stroke, wound complication, bleeding, other morbidity, non-home discharge, and readmission with AUROC’s between 0.87 and 0.96. Given that our ML algorithms perform better than existing tools and logistic regression, they have potential for important utility in the peri-operative management of patients being considered for lower extremity open revascularization to mitigate adverse outcomes and reduce health care costs.

### Supplementary Information

Supplementary Information.

## Data Availability

The data used for this study comes from ACS NSQIP. Access to and use of the data requires approval through an application process available at https://www.facs.org/quality-programs/data-and-registries/acs-nsqip/participant-use-data-file/.

## Code availability

The complete code used for model development and evaluation in this project is publicly available on GitHub: https://github.com/benli12345/LEO-ML-NSQIP.

## References

[1] Zemaitis, M. R., Boll, J. M. & Dreyer, M. A. Peripheral Arterial Disease. in *StatPearls* (StatPearls Publishing, 2021). 28613496

[2] Fowkes FGR (2013). Comparison of global estimates of prevalence and risk factors for peripheral artery disease in 2000 and 2010: A systematic review and analysis. Lancet Lond. Engl..

[3] Agnelli G, Belch JJF, Baumgartner I, Giovas P, Hoffmann U (2020). Morbidity and mortality associated with atherosclerotic peripheral artery disease: A systematic review. Atherosclerosis.

[4] Kim M, Kim Y, Ryu GW, Choi M (2021). Functional status and health-related quality of life in patients with peripheral artery disease: A Cross-sectional study. Int. J. Environ. Res. Public. Health.

[5] Kohn CG, Alberts MJ, Peacock WF, Bunz TJ, Coleman CI (2019). Cost and inpatient burden of peripheral artery disease: Findings from the national inpatient sample. Atherosclerosis.

[6] Farber A (2022). Surgery or endovascular therapy for chronic limb-threatening ischemia. N. Engl. J. Med..

[7] Liang P (2019). In-hospital versus postdischarge major adverse events within 30 days following lower extremity revascularization. J. Vasc. Surg..

[8] Conte MS (2019). Global vascular guidelines on the management of chronic limb-threatening ischemia. J. Vasc. Surg..

[9] Perkins ZB (2020). Predicting the outcome of limb revascularization in patients with lower-extremity arterial trauma: Development and external validation of a supervised machine-learning algorithm to support surgical decisions. Ann. Surg..

[10] Miyata T (2021). Risk prediction model for early outcomes of revascularization for chronic limb-threatening ischaemia. Br. J. Surg..

[11] Biancari F (2007). Risk-scoring method for prediction of 30-day postoperative outcome after infrainguinal surgical revascularization for critical lower-limb ischemia: A Finnvasc registry study. World J. Surg..

[12] Bennett KM, Levinson H, Scarborough JE, Shortell CK (2016). Validated prediction model for severe groin wound infection after lower extremity revascularization procedures. J. Vasc. Surg..

[13] Bilimoria KY (2013). Development and evaluation of the universal ACS NSQIP surgical risk calculator: A decision aid and informed consent tool for patients and surgeons. J. Am. Coll. Surg..

[14] Bertges, D. *et al.* The vascular study group of new england cardiac risk index (VSG-CRI) predicts cardiac complications more accurately than the Revised Cardiac Risk Index in vascular surgery patients. *J. Vasc. Surg.* **52**, (2010). 10.1016/j.jvs.2010.03.031 20570467

[15] Sharma V (2021). Adoption of clinical risk prediction tools is limited by a lack of integration with electronic health records. BMJ Health Care Inform..

[16] Baştanlar Y, Özuysal M (2014). Introduction to machine learning. Methods Mol. Biol..

[17] Shah P (2019). Artificial intelligence and machine learning in clinical development: A translational perspective. NPJ Digit. Med..

[18] Bonde A (2021). Assessing the utility of deep neural networks in predicting postoperative surgical complications: A retrospective study. Lancet Digit. Health.

[19] World Medical Association (2013). World Medical Association Declaration of Helsinki: Ethical principles for medical research involving human subjects. JAMA.

[20] Tfaily MA, Ghanem P, Farran SH, Dabdoub F, Kanafani ZA (2022). The role of preoperative albumin and white blood cell count in surgical site infections following whipple surgery. Sci. Rep..

[21] Collins GS, Reitsma JB, Altman DG, Moons KGM (2015). Transparent Reporting of a multivariable prediction model for individual prognosis or diagnosis (TRIPOD): The TRIPOD statement. Ann. Intern. Med..

[22] ACS NSQIP. *ACS* https://www.facs.org/quality-programs/data-and-registries/acs-nsqip/.

[23] Shiloach M (2010). Toward robust information: Data quality and inter-rater reliability in the American college of surgeons national surgical quality improvement program. J. Am. Coll. Surg..

[24] Cohen ME (2013). Optimizing ACS NSQIP modeling for evaluation of surgical quality and risk: Patient risk adjustment, procedure mix adjustment, shrinkage adjustment, and surgical focus. J. Am. Coll. Surg..

[25] ACS NSQIP Participant Use Data File. *ACS* https://www.facs.org/quality-programs/data-and-registries/acs-nsqip/participant-use-data-file/.

[26] Elfanagely O (2021). Machine learning and surgical outcomes prediction: A systematic review. J. Surg. Res..

[27] Bektaş M, Tuynman JB, Costa Pereira J, Burchell GL, van der Peet DL (2022). Machine learning algorithms for predicting surgical outcomes after colorectal surgery: A systematic review. World J. Surg..

[28] Senders JT (2018). Machine learning and neurosurgical outcome prediction: A systematic review. World Neurosurg..

[29] Chen, T. & Guestrin, C. XGBoost: A Scalable Tree Boosting System. in *Proceedings of the 22nd ACM SIGKDD International Conference on Knowledge Discovery and Data Mining* 785–794 (2016). doi:10.1145/2939672.2939785.

[30] Rigatti SJ (2017). Random forest. J. Insur. Med. N. Y. N.

[31] Zhang Z (2016). Naïve bayes classification in R. Ann. Transl. Med..

[32] Noble WS (2006). What is a support vector machine?. Nat. Biotechnol..

[33] Schmidhuber J (2015). Deep learning in neural networks: an overview. Neural Netw. Off. J. Int. Neural Netw. Soc..

[34] Sperandei S (2014). Understanding logistic regression analysis. Biochem. Medica.

[35] Ngiam KY, Khor IW (2019). Big data and machine learning algorithms for health-care delivery. Lancet Oncol..

[36] Liew BXW, Kovacs FM, Rügamer D, Royuela A (2022). Machine learning versus logistic regression for prognostic modelling in individuals with non-specific neck pain. Eur. Spine J. Off. Publ. Eur. Spine Soc. Eur. Spine Res. Soc..

[37] Shipe ME, Deppen SA, Farjah F, Grogan EL (2019). Developing prediction models for clinical use using logistic regression: An overview. J. Thorac. Dis..

[38] Dobbin KK, Simon RM (2011). Optimally splitting cases for training and testing high dimensional classifiers. BMC Med. Genomics.

[39] Jung Y, Hu J (2015). A K-fold averaging cross-validation procedure. J. Nonparametric Stat..

[40] Adnan M, Alarood AAS, Uddin MI, Ur Rehman I (2022). Utilizing grid search cross-validation with adaptive boosting for augmenting performance of machine learning models. PeerJ Comput. Sci..

[41] Wibowo P, Fatichah C (2022). Pruning-based oversampling technique with smoothed bootstrap resampling for imbalanced clinical dataset of Covid-19. J. King Saud Univ.–Comput. Inf. Sci..

[42] Hajian-Tilaki K (2013). Receiver operating characteristic (ROC) curve analysis for medical diagnostic test evaluation. Casp. J. Intern. Med..

[43] Redelmeier DA, Bloch DA, Hickam DH (1991). Assessing predictive accuracy: How to compare brier scores. J. Clin. Epidemiol..

[44] Loh W-Y, Zhou P (2021). Variable importance scores. . J. Data Sci..

[45] Riley, R. D. *et al.* Calculating the sample size required for developing a clinical prediction model. *BMJ* m441 (2020) 10.1136/bmj.m441. 10.1136/bmj.m441 32188600

[46] Ensor, J., Martin, E. C. & Riley, R. D. Pmsampsize: Calculates the Minimum Sample Size Required for Developing a Multivariable Prediction Model. (2022).

[47] Schafer JL (1999). Multiple imputation: A primer. Stat. Methods Med. Res..

[48] Ross RK, Breskin A, Westreich D (2020). When is a complete-case approach to missing data valid? The importance of effect-measure modification. Am. J. Epidemiol..

[49] Hughes RA, Heron J, Sterne JAC, Tilling K (2019). Accounting for missing data in statistical analyses: Multiple imputation is not always the answer. Int. J. Epidemiol..

[50] R Core Team (2022). R: A language and environment for statistical computing. R Foundation for Statistical Computing, Vienna, Austria. https://www.R-project.org/.

[51] Kuhn, M. *et al.* Caret: Classification and Regression Training. (2022).

[52] Chen T, Guestrin C (2016). XGBoost: A scalable tree boosting system. Proc. 22nd ACM SIGKDD Int. Conf. Knowl. Discov. Data Min.–KDD.

[53] Wright, M. N., Wager, S. & Probst, P. Ranger: A Fast Implementation of Random Forests. (2022).

[54] Naivebayes: High Performance Implementation of the Naive Bayes Algorithm version 0.9.7 from CRAN. https://rdrr.io/cran/naivebayes/.

[55] svm function - RDocumentation. https://www.rdocumentation.org/packages/e1071/versions/1.7-11/topics/svm.

[56] Ripley, B. & Venables, W. Nnet: Feed-Forward Neural Networks and Multinomial Log-Linear Models. (2022).

[57] Robin X (2011). pROC: An open-source package for R and S+ to analyze and compare ROC curves. BMC Bioinformat..

[58] O’Connor DB (2011). An anaesthetic pre-operative assessment clinic reduces pre-operative inpatient stay in patients requiring major vascular surgery. Ir. J. Med. Sci..

[59] Davis FM (2018). The clinical impact of cardiology consultation prior to major vascular surgery. Ann. Surg..

[60] Bonaca MP (2020). Rivaroxaban in peripheral artery disease after revascularization. N. Engl. J. Med..

[61] Conte MS (2015). Society for vascular surgery practice guidelines for atherosclerotic occlusive disease of the lower extremities: Management of asymptomatic disease and claudication. J. Vasc. Surg..

[62] Gerhard-Herman MD (2017). 2016 AHA/ACC guideline on the management of patients with lower extremity peripheral artery disease: Executive summary: A report of the american college of cardiology/American heart association task force on clinical practice guidelines. Circulation.

[63] Aboyans V (2018). Editor’s choice–2017 ESC guidelines on the diagnosis and treatment of peripheral arterial diseases, in collaboration with the European society for vascular surgery (ESVS). Eur. J. Vasc. Endovasc. Surg. Off. J. Eur. Soc. Vasc. Surg..

[64] Stoltzfus JC (2011). Logistic regression: A brief primer. Acad. Emerg. Med. Off. J. Soc. Acad. Emerg. Med..

[65] Kia B (2020). Nonlinear dynamics based machine learning: Utilizing dynamics-based flexibility of nonlinear circuits to implement different functions. PloS One.

[66] Chatterjee P (2019). Nonlinear systems in healthcare towards intelligent disease prediction. Nonlinear Syst.–Theor. Asp. Recent Appl..

[67] Ravaut M (2021). Predicting adverse outcomes due to diabetes complications with machine learning using administrative health data. Npj Digit. Med..

[68] Wang R, Zhang J, Shan B, He M, Xu J (2022). XGBoost machine learning algorithm for prediction of outcome in aneurysmal subarachnoid hemorrhage. Neuropsychiatr. Dis. Treat..

[69] Fang Z-G, Yang S-Q, Lv C-X, An S-Y, Wu W (2022). Application of a data-driven XGBoost model for the prediction of COVID-19 in the USA: A time-series study. BMJ Open.

[70] Viljanen M, Meijerink L, Zwakhals L, van de Kassteele J (2022). A machine learning approach to small area estimation: Predicting the health, housing and well-being of the population of Netherlands. Int. J. Health Geogr..

[71] Gianfrancesco MA, Tamang S, Yazdany J, Schmajuk G (2018). Potential biases in machine learning algorithms using electronic health record data. JAMA Intern. Med..

[72] Mazmudar A, Vitello D, Chapman M, Tomlinson JS, Bentrem DJ (2017). Gender as a risk factor for adverse intraoperative and postoperative outcomes of elective pancreatectomy. J. Surg. Oncol..

[73] Halsey JN, Asti L, Kirschner RE (2022). The impact of race and ethnicity on surgical risk and outcomes following palatoplasty: An analysis of the nsqip pediatric database. Cleft Palate-Craniofac. J. Off. Publ. Am. Cleft Palate.

[74] Rümenapf G, Morbach S, Schmidt A, Sigl M (2020). Intermittent claudication and asymptomatic peripheral arterial disease. Dtsch. Ärztebl. Int..

[75] Shaydakov, M. E. & Tuma, F. Operative Risk. in *StatPearls* (StatPearls Publishing, 2022). 30335273

[76] Bevan GH, WhiteSolaru KT (2020). Evidence-based medical management of peripheral artery disease. Arterioscler. Thromb. Vasc. Biol..

[77] Biscetti F (2021). Outcomes of lower extremity endovascular revascularization: Potential predictors and prevention strategies. Int. J. Mol. Sci..

[78] Roberts DJ (2020). Association between neuraxial anaesthesia or general anaesthesia for lower limb revascularisation surgery in adults and clinical outcomes: Population based comparative effectiveness study. BMJ.

[79] Gillies MA (2017). Intensive care utilization and outcomes after high-risk surgery in Scotland: A population-based cohort study. Br. J. Anaesth..

[80] Patel, P. R. & Bechmann, S. Discharge Planning. in *StatPearls* (StatPearls Publishing, 2022). 32491751

[81] Perez-Riverol Y (2016). Ten simple rules for taking advantage of git and GitHub. PLoS Comput. Biol..

[82] Nguyen LL, Barshes NR (2010). Analysis of large databases in vascular surgery. J. Vasc. Surg..

[83] Northridge ME, Metcalf SS (2016). Enhancing implementation science by applying best principles of systems science. Health Res. Policy Syst..

[84] Bronsert M (2020). Identification of postoperative complications using electronic health record data and machine learning. Am. J. Surg..

[85] Colquhoun DA (2020). Considerations for integration of perioperative electronic health records across institutions for research and quality improvement: The approach taken by the multicenter perioperative outcomes group. Anesth. Analg..

[86] Batko K, Ślęzak A (2022). The use of big data analytics in healthcare. J. Big Data.

[87] Leung SN (2021). Harnessing the full potential of hospital-based data to support surgical quality improvement. BMJ Open Qual..

[88] Eikelboom JW (2017). Rivaroxaban with or without aspirin in stable cardiovascular disease. N. Engl. J. Med..

