# Supplementary material for: Predicting outcomes following lower extremity open revascularization using machine learning
Source: Sci Rep. 2024 Feb 5;14:2899. doi: 10.1038/s41598-024-52944-1 (PMC10844206; doi:10.1038/s41598-024-52944-1)
Supplement: Supplementary file 1 — Supplementary Information. [file 41598_2024_52944_MOESM1_ESM.pdf]

Supplement

Predicting outcomes following lower extremity open revascularization using machine learning

Ben Li MD[1,2,3,4], Raj Verma MD(c)[5], Derek Beaton PhD[6], Hani Tamim PhD[7,8], Mohamad A Hussain MD PhD[9], Jamal J Hoballah MD MBA[10], Douglas S Lee MD PhD[11,12,13], Duminda N Wijeysundera MD PhD[12,13,14,15], Charles de Mestral MD PhD[1,2,12,13,15], Muhammad Mamdani MPH MA PharmD[3,4,6,12,13,15,16], Mohammed Al-Omran MD MSc*[1,2,3,4,8,15,17]

1. Department of Surgery, University of Toronto, Canada
2. Division of Vascular Surgery, St. Michael's Hospital, Unity Health Toronto, University of Toronto, Canada
3. Institute of Medical Science, University of Toronto, Canada
4. Temerty Centre for Artificial Intelligence Research and Education in Medicine (T-CAIREM), University of Toronto, Canada
5. School of Medicine, Royal College of Surgeons in Ireland, University of Medicine and Health Sciences, Ireland
6. Data Science & Advanced Analytics, Unity Health Toronto, University of Toronto, Canada
7. Faculty of Medicine, Clinical Research Institute, American University of Beirut Medical Center, Lebanon
8. College of Medicine, Alfaisal University, Kingdom of Saudi Arabia

9. Division of Vascular and Endovascular Surgery and the Center for Surgery and Public Health, Brigham and Women's Hospital, Harvard Medical School, United States
10. Division of Vascular and Endovascular Surgery, Department of Surgery, American University of Beirut Medical Center, Lebanon
11. Division of Cardiology, Peter Munk Cardiac Centre, University Health Network, Canada
12. Institute of Health Policy, Management and Evaluation, University of Toronto, Canada
13. ICES, University of Toronto, Canada
14. Department of Anesthesia, St. Michael's Hospital, Unity Health Toronto, Canada
15. Li Ka Shing Knowledge Institute, St. Michael's Hospital, Unity Health Toronto, Canada
16. Leslie Dan Faculty of Pharmacy, University of Toronto, Canada
17. Department of Surgery, King Faisal Specialist Hospital and Research Center, Kingdom of Saudi Arabia

***Corresponding author:** Dr. Mohammed Al-Omran, MD, MSc, FRCSC, Department of Surgery, King Faisal Specialist Hospital and Research Center, Riyadh, Saudi Arabia and Division of Vascular Surgery, St. Michael's Hospital, Unity Health Toronto, 30 Bond Street, Suite 7-074, Bond Wing, Toronto, ON, Canada, M5B 1W8.

Telephone: 416-864-6047

Email: mohammed.al-omran@unityhealth.to

Supplementary Table 1. Pre-operative input features for machine learning models

Features (n = 37)	Definition based on ACS NSQIP manual
**Logistics**	
Operation year	Year of procedure
Admission quarter	Quarter of admission
**Demographics**	
Age	Age in years
Sex	Male or female
Body mass index	Weight in kg / height in m^2
Race	As per medical record or self-assigned by the patient: 1) White, 2) Black or African American, 3) American Indian or Alaskan Native, 4) Native Hawaiian or Other Pacific Islander, 5) Asian, 6) Other, 7) Unknown/not reported
Ethnicity	Hispanic or non-Hispanic
Origin status	1) Transferred from another hospital or from 2) home, 3) nursing home, 4) other facility, or 5) unknown
**Comorbidities**	
Hypertension	Diagnosis documented in medical record and patient requires antihypertensive medication within 30 days prior to surgery
Diabetes	Documented diagnosis; non-insulin dependent or insulin-dependent
Current smoker	Patient has smoked cigarettes at any point within the 12 months prior to surgery
Congestive heart failure	Diagnosis documented by physician or advanced provider within 30 days prior to surgery and documentation of at least 1 of the following within 30 days prior to surgery: 1) active signs or symptoms of congestive heart failure, 2) New York Heart Association Functional Classification II-IV, 3) daily prescription of disease-modifying drugs for heart failure, 4) left ventricular ejection fraction < 40% on the most recent measurement prior to surgery, 5) elevated levels of natriuretic peptides (BNP ≥100 pg/mL or NT-proBNP ≥900 pg/mL), 6) patients with one of the following devices: ventricular assist device, implantable cardioverter defibrillator, or cardiac resynchronization therapy device.
Chronic obstructive pulmonary disease	Diagnosis documented in medical record and at least 1 of the following within 30 days prior to surgery: 1) functional disability from chronic obstructive pulmonary disease, 2) requires chronic bronchodilator therapy, 3) hospitalization at any time in the past for treatment of chronic obstructive pulmonary disease, or 4) a forced expiratory volume (FEV) 1 of < 75% on any prior pulmonary function test
Dialysis	Documented peritoneal dialysis, hemodialysis, hemofiltration, hemodiafiltration, or ultrafiltration within 2 weeks prior to surgery

**Features (n = 37)**	**Definition based on ACS NSQIP manual**
Functional status	1) Independent (does not require assistance for activities of daily living), 2) partially dependent (requires some assistance for activities of daily living), 3) totally dependent (requires total assistance for all activities of daily living, or 4) unknown
Physiologic high-risk factor	At least 1 of the following: 1) end stage renal disease, 2) age > 80, 3) New York Heart Association congestive heart failure class III/IV, 4) left ventricular ejection fraction < 30%, 5) unstable angina within 30 days prior to surgery, or 6) myocardial infarction within 30 days prior to surgery
**Medications**	Patient was on medication when considered for surgery or when surgery was decided for the patient
Antiplatelet	Includes aspirin, clopidogrel, eptifibatide, and aggrenox
Statin	Includes atorvastatin, simvastatin, fluvastatin, lovastatin, pitavastatin, rosuvastatin, and pravastatin
Beta blocker	Includes acebutolol, atenolol, betaxolol, bisoprolol, carvedilol, esmolol, labetalol, metoprolol, nadolol, nebivolol, pindolol, propranolol, sotalol, andtimolol
**Pre-operative laboratory investigations**	Collected within 90 days prior to surgery
Serum sodium	Reported in mmol/L
Blood urea nitrogen	Reported in mmol/L
Serum creatinine	Reported in umol/L
Albumin	Reported in g/L
White blood cell count	Reported in cells/mm^3
Hematocrit	Reported in %
Platelet count	Reported in 10^9/L
INR	Unitless ratio
PTT	Reported in seconds
**Anatomy/hemodynamics**	
Limb hemodynamics	1) ABI ≥ 1.3; OR if arteries are described as “noncompressible” AND the toe pressure ≥ 30 mm Hg<br>2) ABI ≥ 1.3; OR if arteries are described as “noncompressible” AND toe pressure < 30 mm Hg<br>3) ABI ≥ 1.30; OR if arteries “noncompressible”, no toe pressure taken<br>4) ABI 0.9- 1.29<br>5) ABI 0.4-0.89<br>6) ABI ≤ 0.39<br>7) ABI not performed AND ipsilateral pedal pulse is palpable<br>8) ABI not performed AND ipsilateral pedal pulse is non-palpable<br>9) None/Not documented
Anatomic high-risk factor	1) Prior ipsilateral bypass involving the currently treated segment, 2) prior ipsilateral endovascular intervention involving the currently treated segment, or 3) none/not documented

Features (n = 37)	Definition based on ACS NSQIP manual
**Concurrent procedures**	Procedure occurring in the same operating room setting as the index surgery
Minor amputation	Amputation below the level of the ankle
Endovascular iliac revascularization	Endovascular revascularization above the level of the common femoral artery
Endovascular infrainguinal revascularization	Endovascular revascularization at the level of the common femoral artery or below
**Other pre-procedural characteristics**	
Symptom status	1) Asymptomatic, 2) claudication, 3) chronic limb threatening ischemia: rest pain, or 4) chronic limb threatening ischemia: tissue loss
Primary procedure	1) Femoropopliteal bypass with single segment saphenous vein,<br>2) Femoropopliteal bypass with prosthetic or spliced vein or composite,<br>3) Femoral distal bypass with single segment saphenous vein,<br>4) Femoral distal bypass with prosthetic or spliced vein or composite,<br>5) Popliteal distal with single segment saphenous vein,<br>6) Popliteal distal bypass with prosthetic or spliced vein or composite, or non-saphenous conduit (such as cephalic vein or cryopreserved vein even if not spliced),<br>7) Femoral endarterectomy, with or without patch angioplasty,<br>8) Profundoplasty,<br>9) Not documented or Other
Urgency	Elective, urgent, or emergent as per documentation by the surgeon, anesthesiologist, nurse, or case schedule
ASA class	1) normal healthy patient, 2) mild systemic disease, 3) severe systemic disease, 4) severe systemic disease that is a constant threat to life, 5) moribund patient who is not expected to survive without the operation, or 6) not reported

Abbreviations: BNP (brain natriuretic peptide), INR (international normalized ratio), PTT (partial thromboplastin time), ABI (ankle brachial index), ASA (American Society of Anesthesiologists)

Supplementary Table 2. Selection of Extreme Gradient Boosting (XGBoost) model hyperparameters using grid search and cross validation

**Hyperparameter**	**Values tested through grid search and cross validation***	**Optimal value chosen to maximize AUROC**
Number of rounds	50, 100, 150, 200, 250, 300, 350, 400, 450, 500	100
Maximum tree depth	2, 3, 4, 5, 6, 7, 8, 9	6
Learning rate	0.4, 0.3, 0.2, 0.1, 0.05, 0.01, 0.001	0.3
Gamma	0, 0.1, 1, 1.5, 2	0
Column sample by tree	0.5, 0.6, 0.7, 0.8, 0.9, 1	1
Minimum child weight	1, 3, 5, 7, 10	1
Subsample	0.5, 0.6, 0.7, 0.8, 0.9, 1	1

*Grid search and cross validation are exhaustive methods that iteratively train and evaluate models using every combination of specified hyperparameter values and selects the set of hyperparameter values that optimize model performance.

Abbreviation: AUROC (area under the receiver operating characteristic curve).

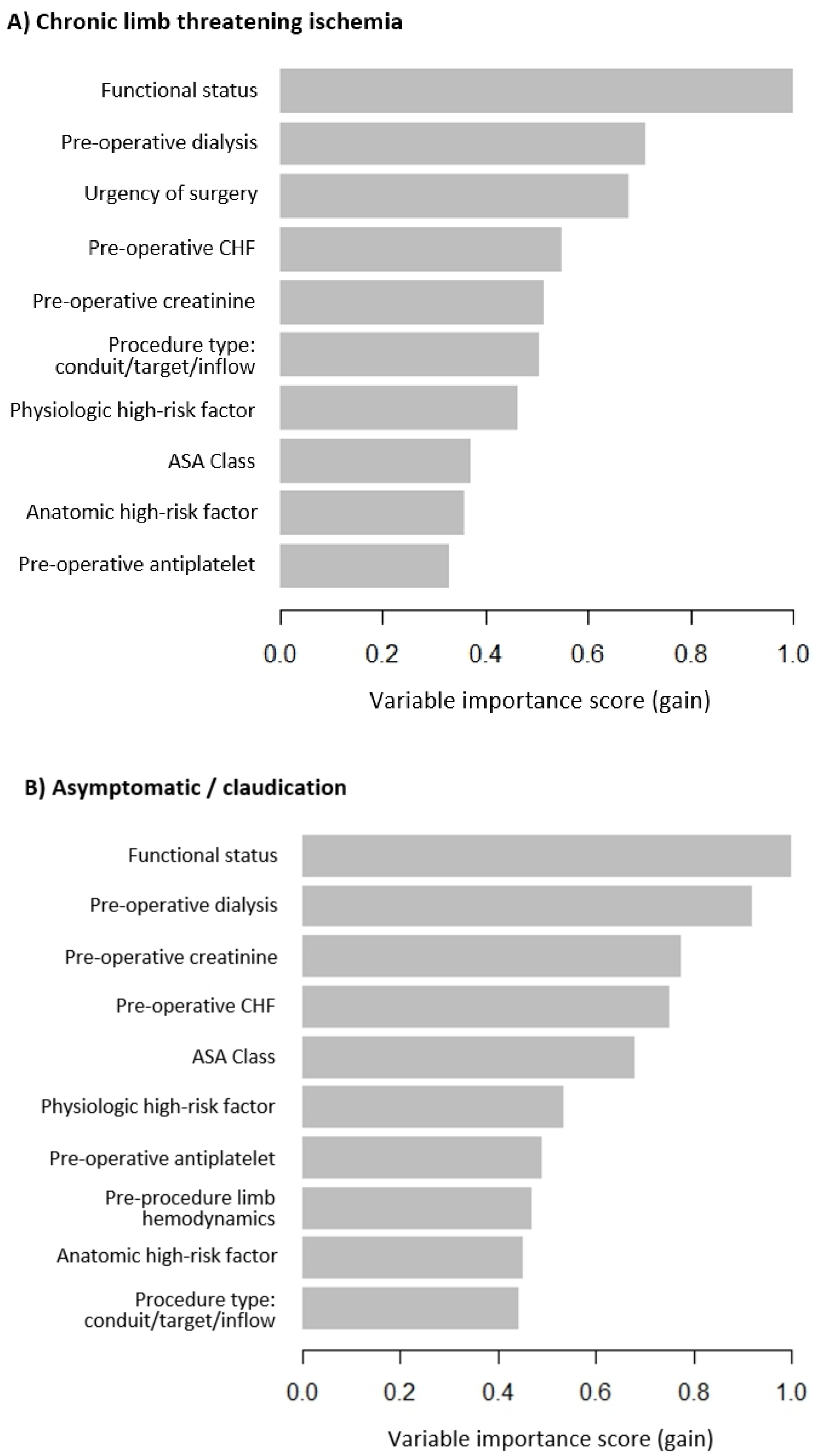

A) Chronic limb threatening ischemia
Functional status
Pre-operative dialysis
Urgency of surgery
Pre-operative CHF
Pre-operative creatinine
Procedure type: conduit/target/inflow
Physiologic high-risk factor
ASA Class
Anatomic high-risk factor
Pre-operative antiplatelet
0.0
0.2
0.4
0.6
0.8
1.0
Variable importance score (gain)
B) Asymptomatic / claudication
Functional status
Pre-operative dialysis
Pre-operative creatinine
Pre-operative CHF
ASA Class
Physiologic high-risk factor
Pre-operative antiplatelet
Pre-procedure limb hemodynamics
Anatomic high-risk factor
Procedure type: conduit/target/inflow
0.0
0.2
0.4
0.6
0.8
1.0
Variable importance score (gain)

Supplementary Figure 1. Variable importance scores for the top 10 predictors of 30-day major adverse limb event or death following lower extremity open revascularization in the Extreme Gradient Boosting (XGBoost) model with subgroup analysis based on symptom status: A) chronic limb threatening ischemia and B) asymptomatic / claudication. Abbreviations: CHF (congestive heart failure), ASA (American Society of Anesthesiologists).

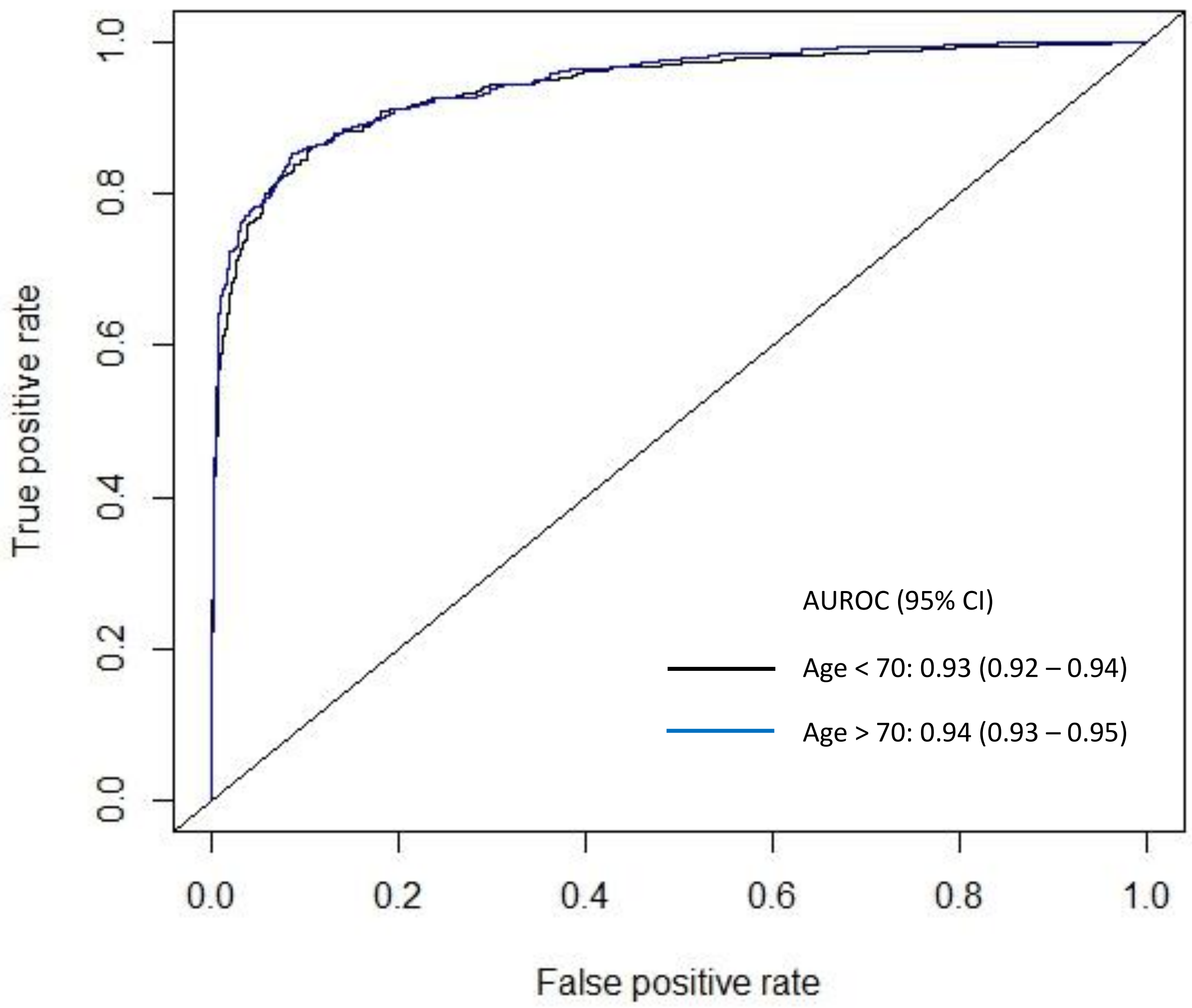

Supplementary Figure 2. Receiver operating characteristic curve for 30-day major adverse limb event or death following lower extremity open revascularization using Extreme Gradient Boosting (XGBoost) model with subgroup analysis based on age. AUROC (area under the receiver operating characteristic curve), CI (confidence interval).

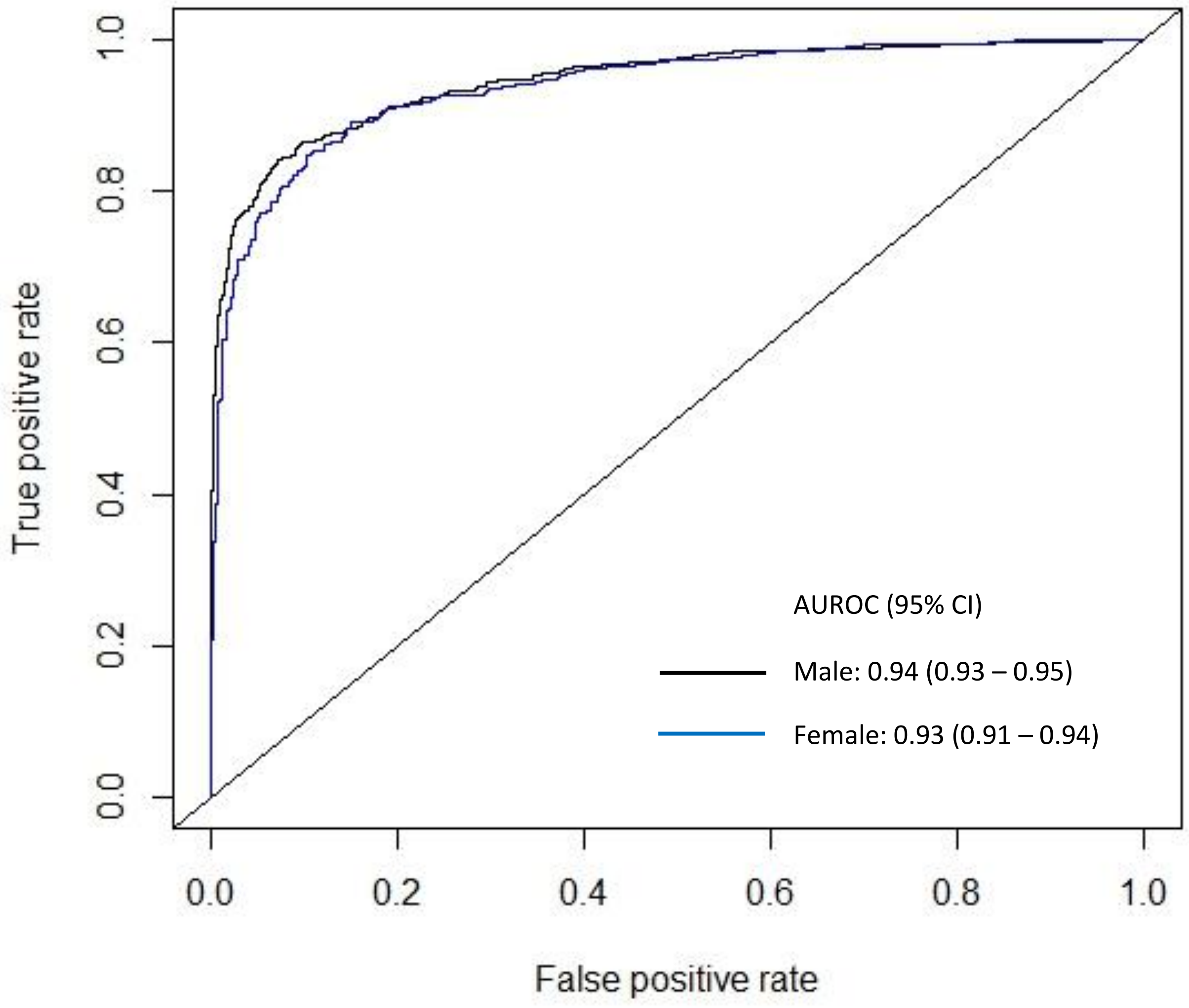

Supplementary Figure 3. Receiver operating characteristic curve for predicting 30-day major adverse limb event or death following lower extremity open revascularization using Extreme Gradient Boosting (XGBoost) model with subgroup analysis based on sex. AUROC (area under the receiver operating characteristic curve), CI (confidence interval).

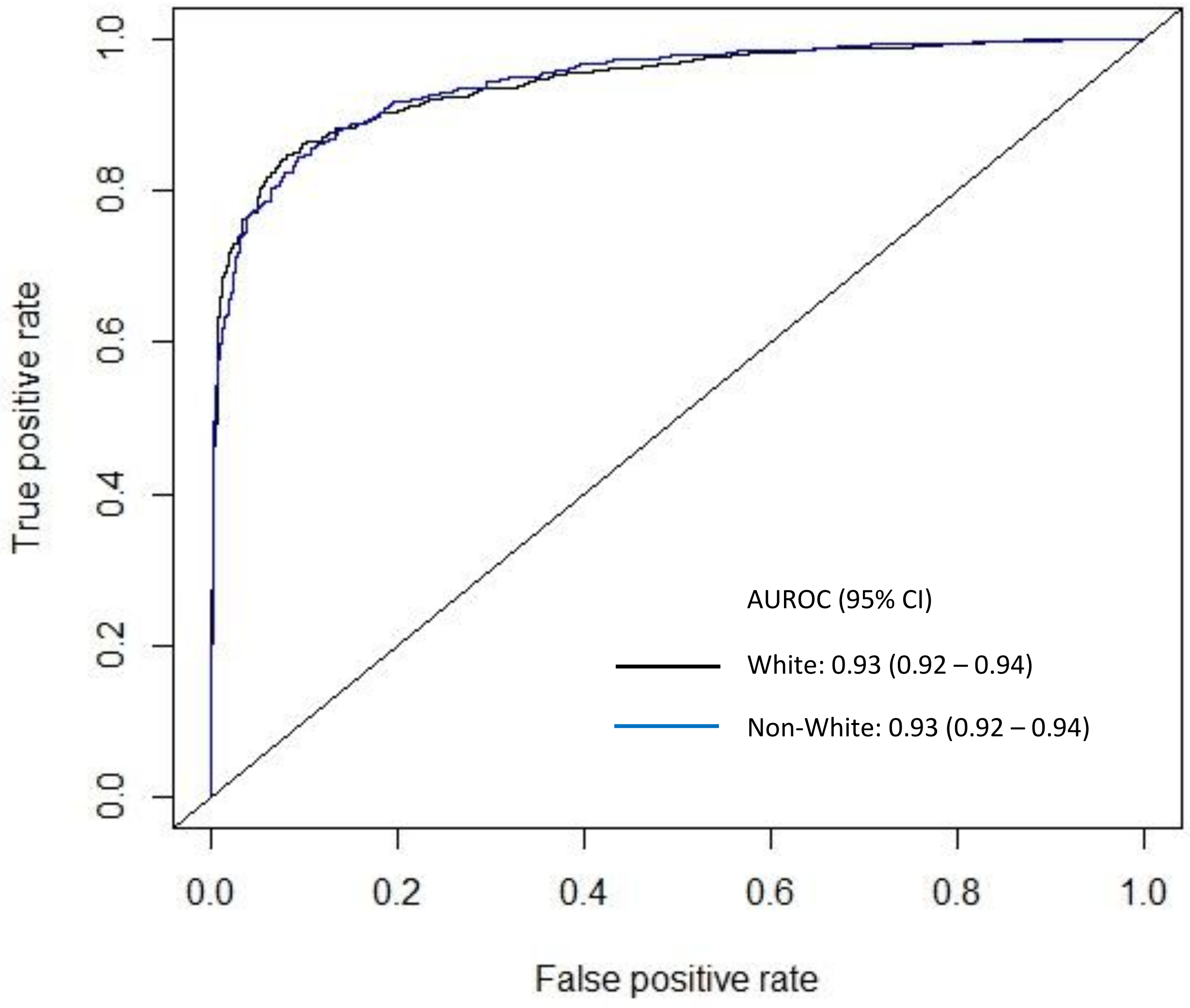

Supplementary Figure 4. Receiver operating characteristic curve for predicting 30-day major adverse limb event or death following lower extremity open revascularization using Extreme Gradient Boosting (XGBoost) model with subgroup analysis based on race. AUROC (area under the receiver operating characteristic curve), CI (confidence interval).

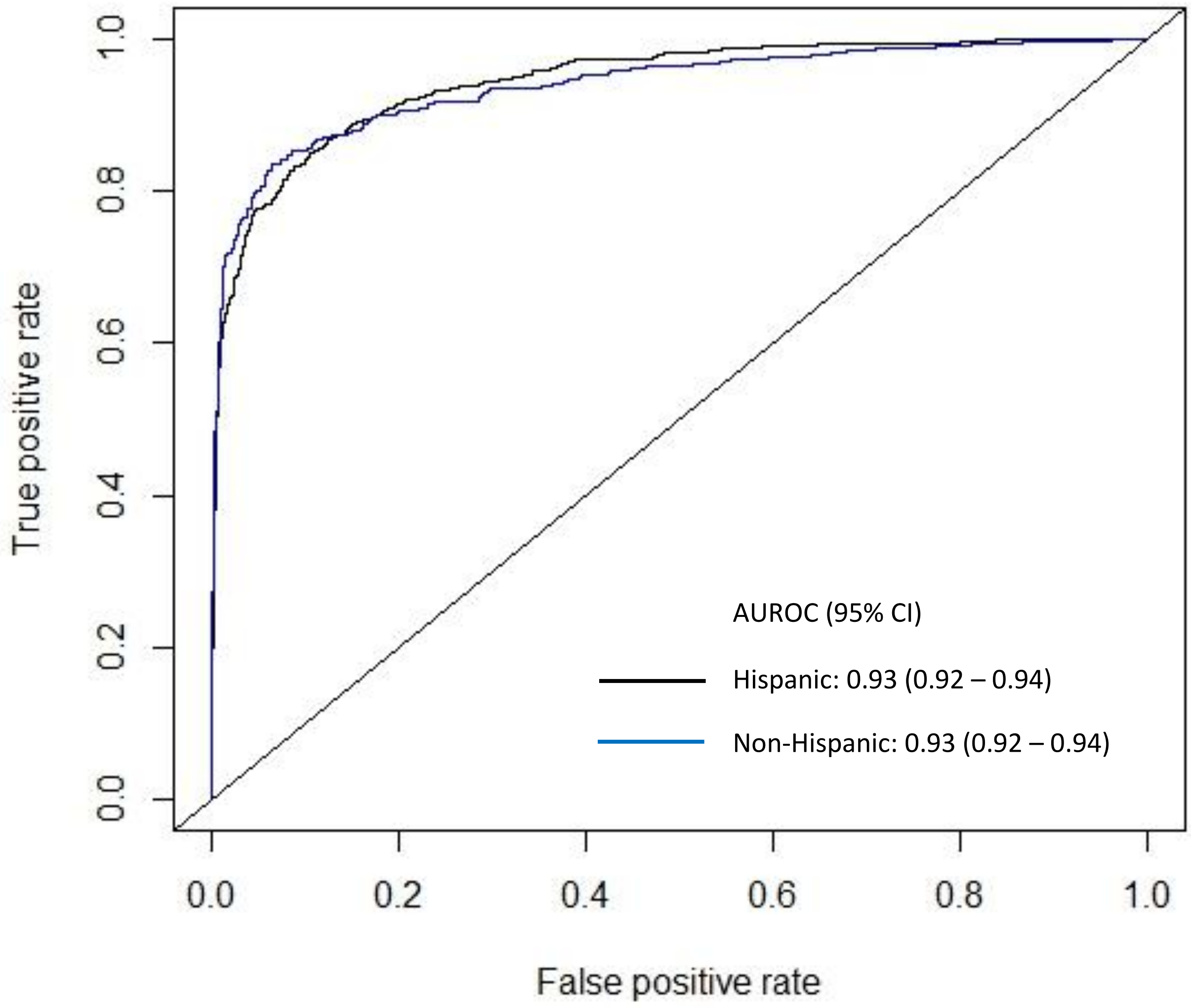

Supplementary Figure 5. Receiver operating characteristic curve for predicting 30-day major adverse limb event or death following lower extremity open revascularization using Extreme Gradient Boosting (XGBoost) model with subgroup analysis based on ethnicity. AUROC (area under the receiver operating characteristic curve), CI (confidence interval).

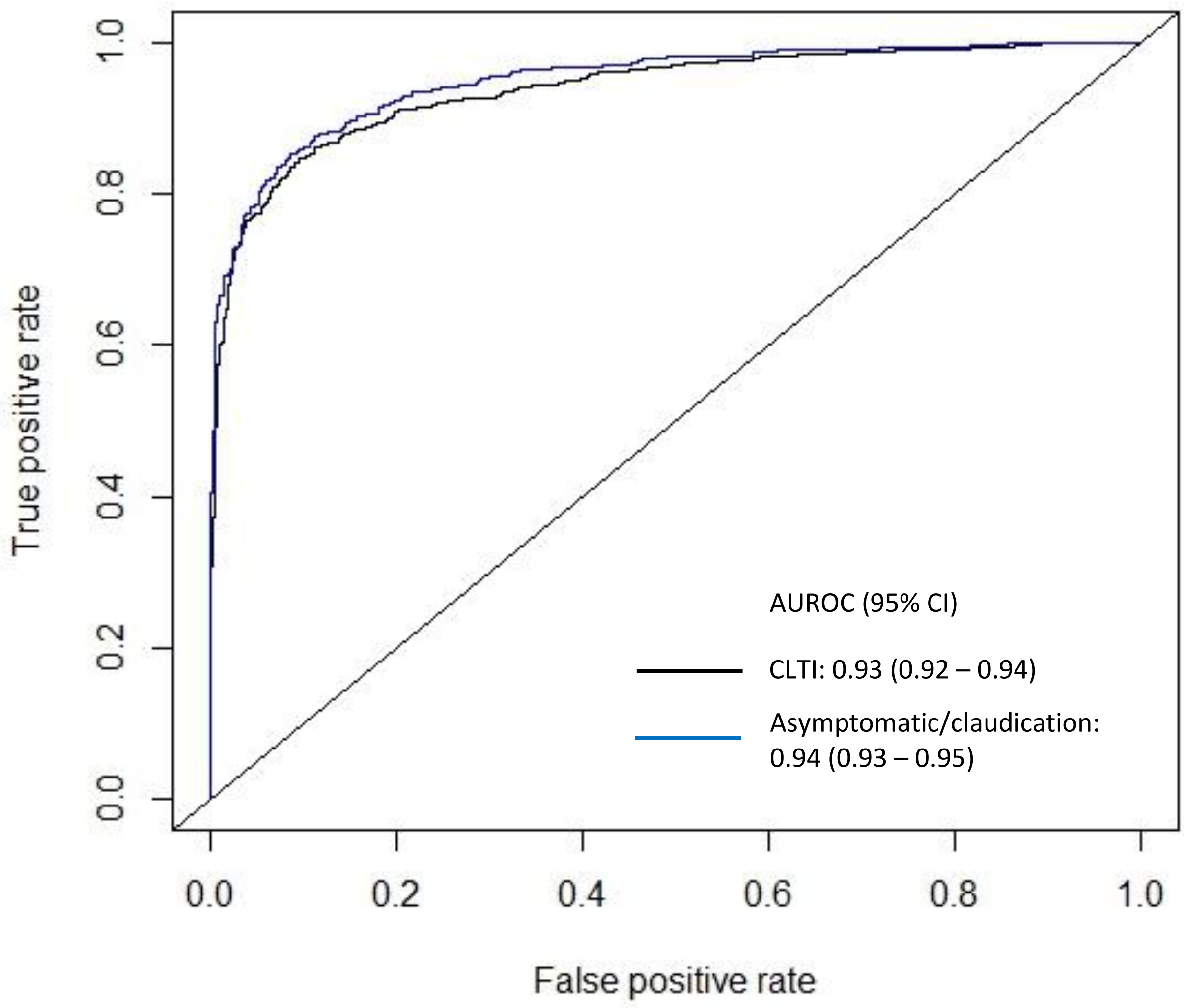

Supplementary Figure 6. Receiver operating characteristic curve for predicting 30-day major adverse limb event or death following lower extremity open revascularization using Extreme Gradient Boosting (XGBoost) model with subgroup analysis based on symptom status. AUROC (area under the receiver operating characteristic curve), CI (confidence interval), CLTI (chronic limb threatening ischemia).

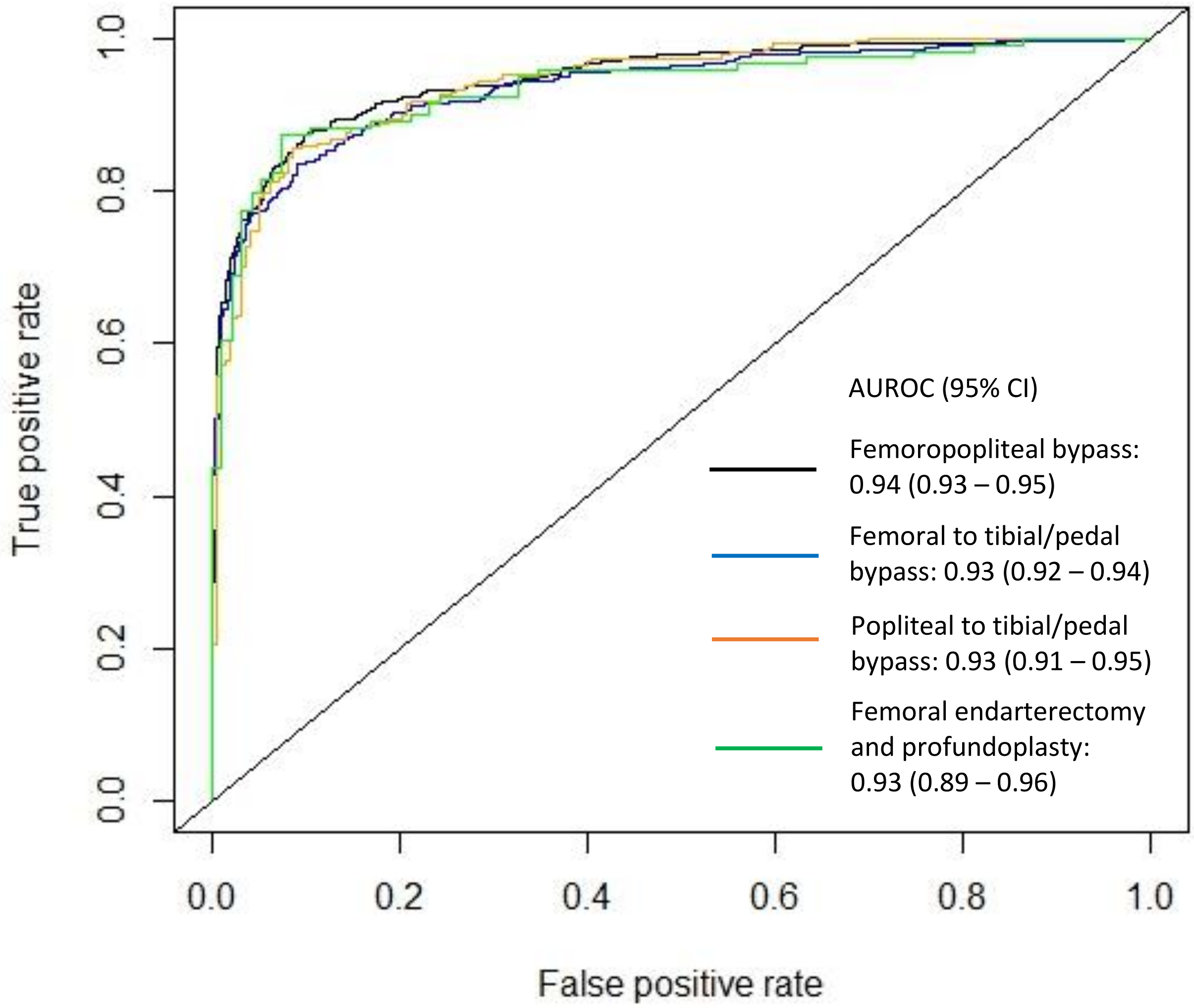

Supplementary Figure 7. Receiver operating characteristic curve for predicting 30-day major adverse limb event or death following lower extremity open revascularization using Extreme Gradient Boosting (XGBoost) model with subgroup analysis based on procedure type. AUROC (area under the receiver operating characteristic curve), CI (confidence interval).

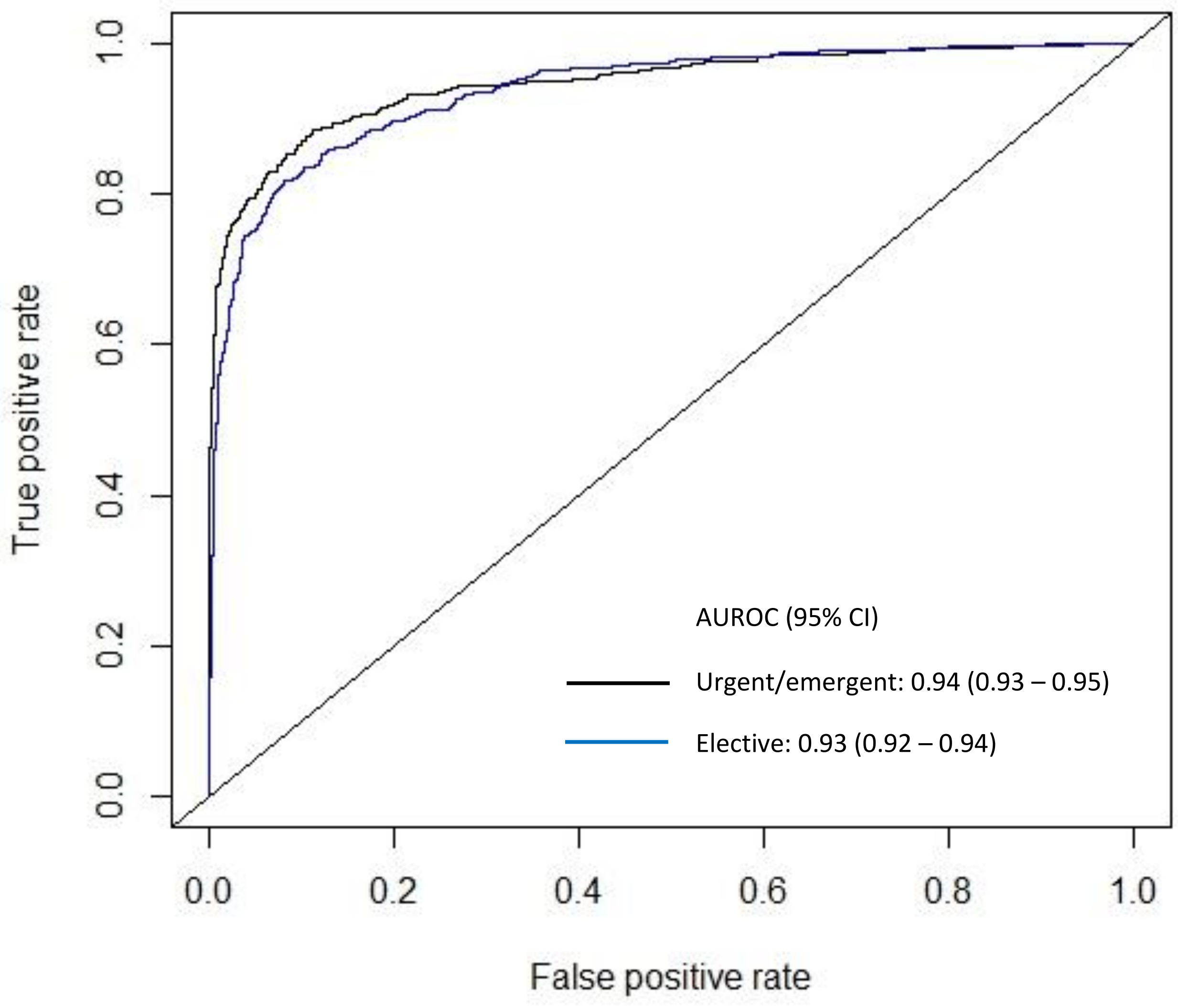

Supplementary Figure 8. Receiver operating characteristic curve for predicting 30-day major adverse limb event or death following lower extremity open revascularization using Extreme Gradient Boosting (XGBoost) model with subgroup analysis based on urgency of surgery. AUROC (area under the receiver operating characteristic curve), CI (confidence interval).
